# An alternatively spliced form affecting the Marked Box domain of *Drosophila* E2F1 is required for proper cell cycle regulation

**DOI:** 10.1371/journal.pgen.1007204

**Published:** 2018-02-08

**Authors:** Minhee Kim, Jack P. Tang, Nam-Sung Moon

**Affiliations:** Department of Biology, Developmental Biology Research Initiative, McGill University, Montreal, Quebec, Canada; Geisel School of Medicine at Dartmouth, UNITED STATES

## Abstract

Across metazoans, cell cycle progression is regulated by E2F family transcription factors that can function as either transcriptional activators or repressors. For decades, the *Drosophila* E2F family has been viewed as a streamlined RB/E2F network, consisting of one activator (dE2F1) and one repressor (dE2F2). Here, we report that an uncharacterized isoform of dE2F1, hereon called dE2F1b, plays an important function during development and is functionally distinct from the widely-studied dE2F1 isoform, dE2F1a. dE2F1b contains an additional exon that inserts 16 amino acids to the evolutionarily conserved Marked Box domain. Analysis of *de2f1b*-specific mutants generated via CRISPR/Cas9 indicates that dE2F1b is a critical regulator of the cell cycle during development. This is particularly evident in endocycling salivary glands in which a tight control of dE2F1 activity is required. Interestingly, close examination of mitotic tissues such as eye and wing imaginal discs suggests that dE2F1b plays a repressive function as cells exit from the cell cycle. We also provide evidence demonstrating that dE2F1b differentially interacts with RBF1 and alters the recruitment of RBF1 and dE2F1 to promoters. Collectively, our data suggest that dE2F1b is a novel member of the E2F family, revealing a previously unappreciated complexity in the *Drosophila* RB/E2F network.

## Author summary

The *Drosophila* E2F1 (dE2F1) protein has been studied as one of the principal regulators of cell cycle control in both mitotic cells and cells undergoing a variant cell cycle called the endocycle. dE2F1 is the sole “activator” E2F of the highly streamlined *Drosophila* RB/E2F network. However, there has been evidence suggesting that this simplistic view of the activator E2F may not be true and that dE2F1 can also provide a repressive function. Elucidating the dual role of dE2F1 in transcriptional regulation has been elusive. In our report, we investigate an uncharacterized isoform of dE2F1 that we have termed as dE2F1b. Notably, the evolutionarily conserved Marked Box domain, which is important for target specificity and protein-protein interactions, is altered in this isoform. Our findings suggest that dE2F1b is required for proper cell cycle control in both mitotic and endocycling cells. Strikingly, we show that dE2F1b has repressive functions in a context-dependent manner. Overall, our findings reveal an unanticipated complexity to dE2F1, providing important insights into the dual function of dE2F1 in transcriptional regulation.

## Introduction

The E2F family of transcription factors was first cloned as a cellular factor that binds to the Early E2 region of the adenovirus genome [1]. Since its discovery, families of E2F transcription factors have been identified in metazoans ranging from nematodes to mammals [2]. One of the important features of E2F transcription factors is their ability to bind to a consensus sequence, TTTCCCGC, which is commonly found in cell cycle-regulated genes [3, 4]. While most E2Fs heterodimerize with DP to bind the consensus sequence, a subset of E2F proteins bind DNA without the help of DP [5]. Regardless, the ability of multiple members of E2F to bind and regulate the same gene allows fine regulation of target gene expression during development [2].

E2F family proteins can be classified as either “activators” or “repressors” based on their role in transcription [6]. This classification was largely attributed by studies in fruit flies, which have only two E2F genes, *de2f1* and *de2f2*. Genetic and biochemical assays demonstrated that dE2F1 and dE2F2 have antagonistic functions [7]. While both can directly bind to a common set of genes, dE2F1 promotes whereas dE2F2 represses transcription [8]. As a consequence, the phenotype observed in *de2f1* mutant flies can be largely suppressed by inactivating dE2F2 [7]. The simplicity of the *Drosophila* E2F network provided an example in which the interplay between activator and repressor E2Fs coordinates target gene expression and cell cycle progression. Curiously, while the anti-proliferative effect of dE2F2 was clearly demonstrated in the *de2f1* mutant background, *de2f2* mutant flies do not display a strong cell cycle defect [7, 9]. This suggests that in the presence of dE2F1, dE2F2’s role as a repressor is largely dispensable. Notably, there has been evidence suggesting a repressive role for dE2F1 during development, although the molecular mechanism underling this observation has been elusive [10, 11].

In mammals, E2F1, E2F2 and E2F3 are considered to be activator E2Fs while E2F4 to 8 are considered to be repressor E2Fs [2]. Since they have similar DNA-binding specificities, functional redundancies within activator and repressor E2Fs clearly exist. Indeed, all three activator E2Fs must be inactivated in mice to completely inhibit E2F-dependent transcription program and cellular proliferation [12]. In addition, a study by Tsai *et al*. demonstrated that activator E2Fs can compensate for each other’s function as long as their expression is properly controlled [13]. Specifically, Tsai *et al* generated mice expressing *E2F1* under the control of the *E2F3* promoter in the *E2F1* to *3* triple-mutant background. These mice are phenotypically similar to *E2F1* and *E2F2* double knockout mice and not to *E2F2* and *E2F3* double knockout mice. This study elegantly demonstrated that it is the expression pattern, not the protein sequence, that determines the functional specificity of activator E2Fs during mouse development.

Despite the genetic evidence showing similarities between the *in vivo* functions of activator E2Fs, member-specific activities among mammalian activator E2Fs have been identified. A key molecular difference between activator E2Fs resides in the Marked Box (MB) domain. Domain swapping experiments coupled with transcriptome analysis revealed that the MB domain is responsible for generating E2F1- and E2F3-specific gene signatures [14]. This finding was further supported by the observation that the MB domain mediates specific protein-protein interactions. For example, E2F3 was shown to specifically interact with an E-box transcription factor, TFE3, through the MB domain to cooperatively regulate target gene expression [15]. The MB domain was also shown to be important for the physical interaction between E2Fs and the C-terminal (C-term) domain of RB family proteins in mammals [16–18]. Structural studies have identified amino acid residues that are important for member-specific interaction between E2F and RB family proteins. Interestingly, these residues include the key amino acids that mediate an E2F1-specific interaction with the RB tumor suppressor protein, pRB, that allows the pRB/E2F1 complex to silence repetitive sequences [19, 20]. Overall, the MB domain plays a crucial function, allowing members of activator E2Fs to carry out specific functions.

**Fig 1:**
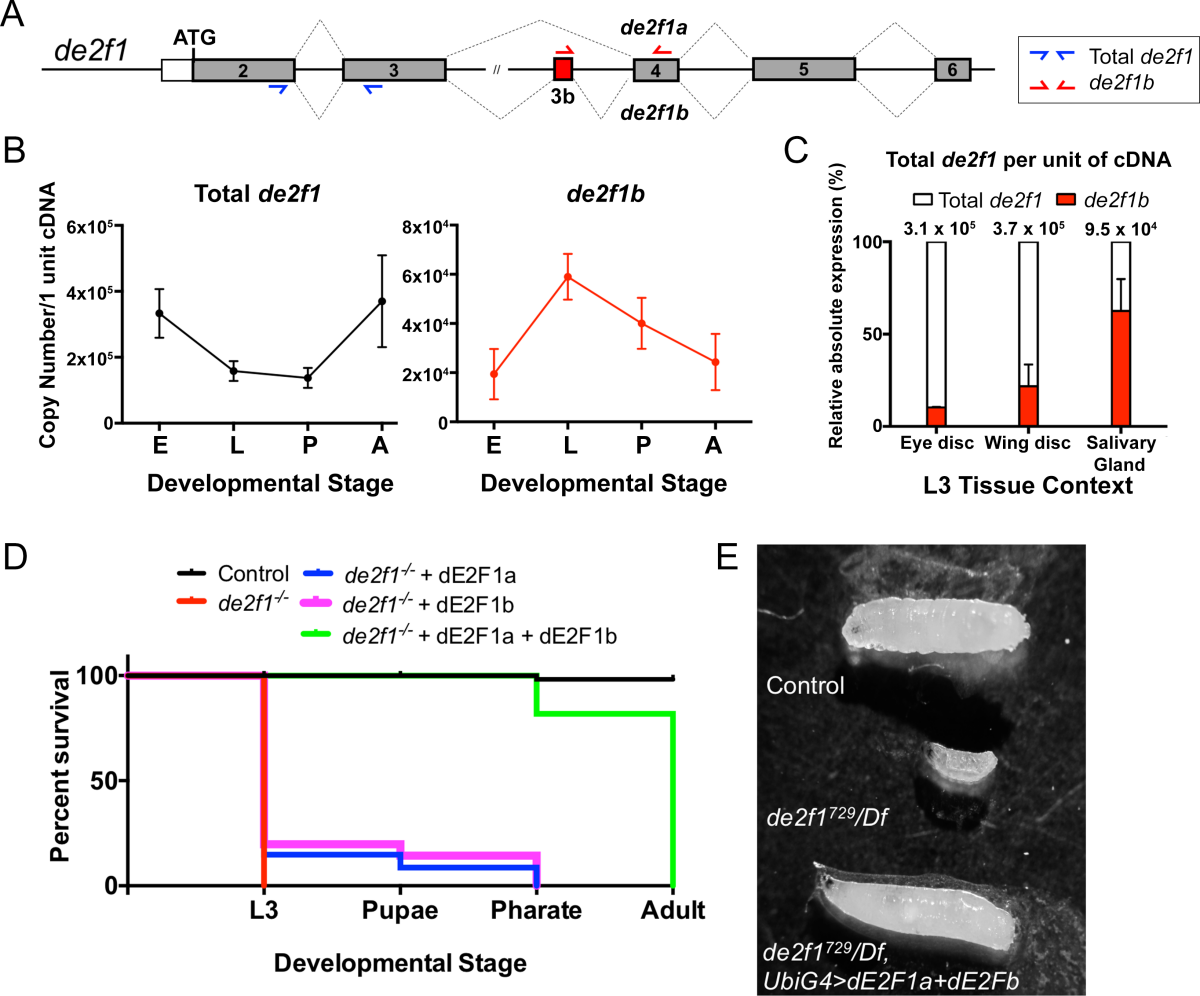
Two alternatively spliced forms of *de2f1*, *def1a* and *de2f1b*, are required to rescue larval lethality of *de2f1* mutant flies. (A) A schematic of the coding region of the *de2f1* gene showing the two alternatively spliced forms of *de2f1*, *de2f1a* and *de2f1b*. The exon highlighted in red indicates the *de2f1b*-specific exon, exon 3b. Primer locations for absolute quantification of total *de2f1* and *de2f1b* are indicated in blue and red, respectively. (B) RT-qPCR is performed to estimate the absolute levels of the total *de2f1* RNA and *de2f1b-*specific RNA at different developmental stages (E: embryonic, L: larval, P: pupal and A: adult). The y-axis indicates the copy number per 1 unit of cDNA (1 unit of cDNA represents 25 ng of RNA). The error bars indicate standard error of the mean (s.e.m.) of triplicated independent biological replicates. (C) The copy numbers of the total *de2f1* RNA and *de2f1b-*specific RNA in indicated third instar larval tissues are determined. The graph shows the percentage contribution of the *de2f1b-*specific transcript to the total *de2f1*. Error bars indicate s.e.m. (D) Molecular complementation tests are performed in *de2f1* mutants by expressing either *de2f1a* or *de2f1b* alone or by expressing both. A survival curve from third instar larval stage (L3) to adult of indicated genotypes is presented. The percent survival is determined by comparing the observed frequency of survival to the expected frequency based on Mendelian ratio (see materials and methods). (E) A control (*yw*), *de2f1* mutant (*de2f1*^*729*^*/Df*) and rescued (*de2f1*^*729*^*/Df*, *UbiG4>dE2F1+dE2Fb*) larvae are shown.

We recently reported that the fruit fly activator *E2F*, *de2f1*, is transcribed from multiple transcription start sites, and that specific promoters are required for proper cell cycle exit during development [21]. Interestingly, the annotated sequences show that an alternatively spliced isoform of *de2f1* is associated with the promoter we identified (http://flybase.org/reports/FBgn0011766.html). The alternatively spliced form, hereon referred as *de2f1b*, contains an extra exon that adds 48 nucleotides to the *de2f1* isoform that has been widely studied for more than two decades, hereon referred as *de2f1a* (Fig 1A). Notably, the additional exon of *de2f1b* alters the amino acid sequences within the MB domain. In this study, we provide evidence that dE2F1b is a novel member of the *Drosophila* E2F family that is functionally distinct from dE2F1a. We demonstrate that dE2F1b is an essential isoform of dE2F1 through molecular complementation tests. While neither dE2F1a nor dE2F1b alone is sufficient to efficiently rescue the early larval lethality of *de2f1* mutants, co-expression of both isoforms is. In addition, generation of a *de2f1b*-specific mutant via CRISPR/Cas9 revealed that dE2F1b is a critical regulator of the cell cycle. We also provide evidence to suggest that dE2F1b is required to negatively regulate E2F target gene expression in specific contexts although dE2F1 has been classified as an activator E2F. Overall, our study identifies dE2F1b as a novel member of E2F and reveals a previously unappreciated complexity in the *Drosophila* RB/E2F network.

## Results

The alternatively spliced isoform of *de2f1*, *de2f1b*, contains an extra exon, exon 3b, that adds 48 nucleotides to the widely studied *de2f1* isoform, *de2f1a* (Fig 1A). As a first step to gain insights into the biological function of *de2f1b*, we determined the expression level of total *de2f1* and *de2f1b*-specific transcripts at different developmental stages. We performed quantitative RT-PCR (RT-qPCR) using RNA isolated from embryos, larvae, pupae, and adult flies, then calculated the copy number per unit of cDNA (see materials and methods). The copy number of total *de2f1* per unit of cDNA ranges from 1.4 X 10^5^ to 3.7 X 10^5^, the lowest being at the pupal stage and the highest being at the adult stage (Fig 1B). The *de2f1b* transcript is expressed at a lower level, ranging from 1.6 X 10^4^ to 5.6 X 10^4^ copies per unit of cDNA. Interestingly, the expression of *de2f1b* peaks at the larval stage, 5.6 X 10^4^ copies, when the total *de2f1* level is relatively low, 1.6 X 10^5^ copies. Therefore, the *de2f1b-*isoform represents approximately one third of the total *de2f1* at the larval stage, the highest of all stages. We also determined the relative expression level of total *de2f1* and *de2f1b* transcripts in several third instar larval (L3) tissues (Fig 1C). Notably, 5.3 X 10^4^ copies of the *de2f1b* isoform is expressed in salivary glands, representing 56% of the total *de2f1*. In eye and wing imaginal discs, *de2f1b* represents less than 20% of total *de2f1*. Taken together, these results suggest that *de2f1b* expression is developmentally regulated and contributes to a significant fraction of the total *de2f1* transcript the salivary gland.

Because the only difference between dE2F1a and dE2F1b is the 16 amino acids coded by exon 3b (Fig 1A), we were unable to directly detect dE2F1b at the protein level. Our efforts to raise a peptide antibody against the 16 amino acids did not yield a functional antibody. Nevertheless, we asked whether dE2F1b plays a specific role during development by performing a molecular complementation test in a *de2f1* mutant background. *de2f1* mutants have severe developmental defects and mostly die at the early larval stage [22, 23]. We expressed either *de2f1a* or *de2f1b* alone or together in a *de2f1* mutant background and determined their ability to rescue the early larval lethality (Fig 1D). *de2f1a* or *de2f1b* expression alone produces third instar larvae at a much lower frequency than the expected ratio. In addition, the rescued animals are developmentally delayed, only progressing to third instar larvae 10 days after egg laying (S1C Fig). Strikingly, expression of both isoforms results in third instar larvae at the expected frequency with only one day of developmental delay (Fig 1D and 1E). Moreover, a majority of the rescued third instar larvae complete pupariation and became pharate adults (S1A and S1B Fig) while none of *de2f1a* or *de2f1b* alone larvae progress to pharate adults. Importantly, doubling the amount of *de2f1a* by expressing two copies of the transgene results in only 23.1% of survival at the third larval stage and all surviving larvae fail to progress to pharate adults (S1D Fig). These results indicate that it is the presence of both isoforms that is important for rescue, and not the dosage of the transgenes. Overall, the molecular complementation tests indicate that dE2F1b is a functionally important isoform of dE2F1 and that dE2F1a and dE2F1b likely play distinct roles during development.

**Fig 2:**
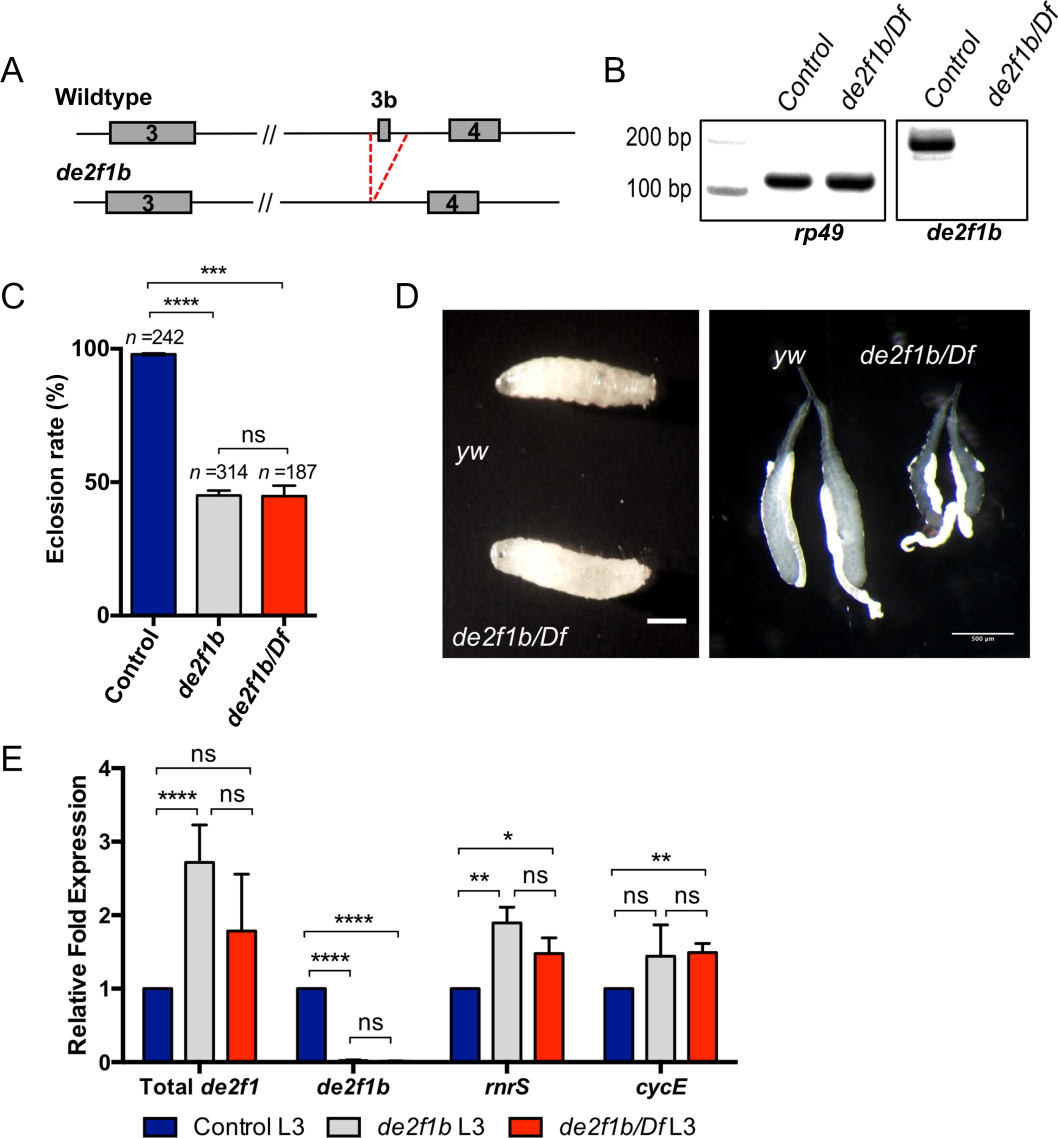
*de2f1b*-specific mutant flies have reduced viability. (A) A schematic of the *de2f1* gene region shows the deletion of the 3b exon in *de2f1b* mutant flies. (B) RT-PCR confirms the lack of *de2f1b* transcript in the trans-heterozygous flies between *de2f1b* mutant and a deficiency line covering the *de2f1b* locus (*de2f1b/Df*). *yw* flies were used as control. (C) Quantification of the eclosion rate of the *de2f1b* mutants is shown. *de2f1b* homozygous (*de2f1b/de2f1b)* and trans-heterozygous over a deficiency (*de2f1b/Df*) flies are compared to a control (*yw*). The error bars indicate standard deviation (s.d.) of three independent experiments and the number of total pupae examined is indicated. (D) A control (*yw*) and *de2f1b* (*de2f1b/Df*) third instar larvae are shown on the left and their salivary glands are shown on the right. The scale bar for larvae is 1mm and for salivary glands is 0.5 mm. (E) Relative levels of the total *de2f1*, *de2f1b*, *CycE and rnrS* transcripts are determined by RT-qPCR. RNA samples are isolated from control (*yw*) and *de2f1b* mutants (*de2f1b* and *de2f1b/Df*) third instar larvae. The error bars indicate s.d. of triplicated biological replicates. Statistical testing in 2C and 2E were performed using two-tailed t-tests where ns = p>0.5; * = p ≤ 0.05; ** = p ≤ 0.01; *** = p ≤ 0.001; **** = p≤ 0.0001.

To determine the *in vivo* function of *de2f1b*, a *de2f1b*-specific mutant line was generated using the CRISPR/Cas9 system. This was achieved by targeted removal of the *de2f1b*-specific exon and its surrounding splicing acceptor and donor sites (Fig 2A). The *de2f1b* mutant line was validated by sequencing of its genomic DNA (S2A Fig) and through RT-PCR to verify the absence of exon 3b (Fig 2B). In addition, cDNA from *de2f1b* mutants was sequenced to confirm that this deletion does not result in a frameshift mutation in *de2f1a* (S2C Fig). At the organismal level, homozygous *de2f1b* mutant flies develop normally with no apparent defects until the late pupal stage where about 50% of *de2f1b* mutants fail to complete metamorphosis and die prior to eclosion (Fig 2C). Similar eclosion rates were observed in both *de2f1b* homozygous mutants and trans-heterozygous mutants of *de2f1b* and a deficiency covering *de2f1*, indicating that this phenotype is specific to the *de2f1b* mutation. We also observed that *de2f1b* mutants are female sterile but male fertile, indicating an ovary-specific defect. Indeed, ovaries from well-fed five days old *de2f1b* females are dramatically smaller than the control (S2B Fig). At the late third instar larval stage (105–110 hrs after egg laying, AEL), while the sizes of *de2f1b* and control L3 larvae are comparable, the overall size of the *de2f1b* salivary gland is consistently smaller than the control (Fig 2D). This observation together with the expression data shown in Fig 1C suggests dE2F1b plays a critical function during salivary gland development. To determine the effect of *de2f1b* mutation on the overall level of *de2f1* transcript, RT-qPCR was performed using RNA isolated from third instar larvae (Fig 2E). Surprisingly, the total level of *de2f1* is increased in *de2f1b* mutant larvae and the expression of E2F target genes, *cyclin E* (*cycE*) and *ribonucleotide reductase small subunit* (*rnrS*), is also increased in *de2f1b* mutant larvae. Overall, the *de2f1b*-specific mutation results in tissue specific defects and an overall increase in dE2F1 expression/activity during development.

**Fig 3:**
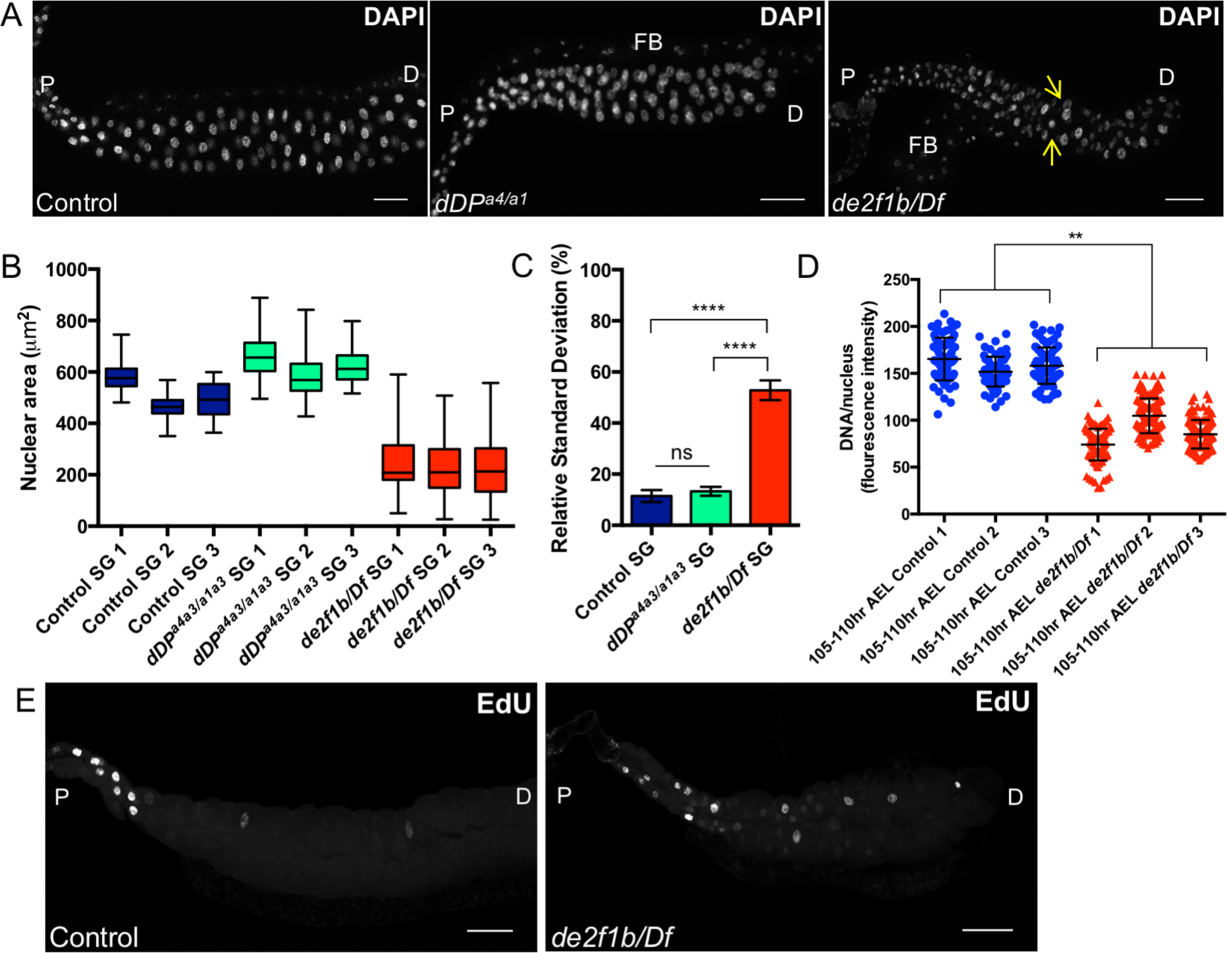
*de2f1b* mutant salivary glands display disrupted endocycle progression. (A) Late stage (105–110 hour After Egg Laying, AEL) salivary glands from a control, *dDP* and *de2f1b* mutant larvae are stained with DAPI to visualize nuclei. Yellow arrows show an example of nuclei with different sizes. (B) A box and whisker graph showing the distribution of nuclear area (μm^2^) in control, *dDP* and *de2f1b* mutant salivary glands is presented. Three salivary glands are used for each genotype. (C) Average of the relative standard deviation of nuclear area of indicated genotype is shown. Values represent the mean of triplicated biological replicates and error bars represent s.d. One-way ANOVA was used to calculate statistical significance, where ns = p>0.05; *** = p≤0.001. (D) Scatter plot representing DAPI intensity from 105–110 hour AEL salivary glands from indicated genotypes. The values in the y-axis represent fluorescence intensity of each nuclei. Average mean fluorescence intensity was compared between control group (1–3) and *de2f1b* mutant group (1–3). ** = p ≤ 0.01 using two-tailed t-test. (E) Salivary glands of indicated genotype are labeled with EdU to visualize S-phase cells. Scale bars for all salivary glands indicate 100 μm. P: proximal end. D: distal end. FB: Fat body.

We next examined the *de2f1b* mutant salivary gland in closer detail. The *Drosophila* larval salivary gland is an endoreplicating tissue where cells undergo a dramatic period of growth by increasing in ploidy through a variant cell cycle called the endocycle [24]. It consists of repeated cycles of G1 and S phases without intervening mitoses. While other *Drosophila* tissues grow by endocycle, L3 salivary glands are exceptional in that their cells contain more than 1000 copies of their genome in the form of polytene chromosomes [25]. To determine whether the size defects observed in Fig 2D is related to DNA content, we visualized nuclei using 4′,6-Diamidine-2′-phenylindole dihydrochloride (DAPI). During larval growth, the salivary glands grow from the distal-tip to the proximal-end in a coordinated fashion [26]. In control late-stage (105–110 hr AEL) L3 salivary glands, this coordinated growth is apparent since the nuclear sizes are similar across a single tissue (Fig 3A). However, in late-stage *de2f1b* salivary glands, a striking variation of nuclear size was observed (Fig 3A yellow arrows). Quantification of the overall distribution of nuclear area of three individual salivary glands of control and *de2f1b* mutants indicates that, on average, nuclear sizes are smaller and are significantly variable in *de2f1b* mutants than in control salivary glands (Fig 3B and 3C). Notably, quantification of DAPI intensity revealed that *de2f1b* salivary glands have nuclei with lower DNA content on average than the control (Fig 3D). This result indicates that the smaller nuclear sizes observed in *de2f1b* salivary glands likely reflects lower DNA content. We next determined the pattern of S-phase cells in late-stage *de2f1b* salivary glands using ethynyl deoxyuridine (EdU) labeling, a thymidine analog. While control late-stage L3 salivary glands have S-phase cells prominently in the proximal region, S-phase cells with variable intensities of EdU are found throughout *de2f1b* late-stage L3 salivary glands (Fig 3E). This result indicates a failure to properly control endocycle progression in *de2f1b* salivary glands. Importantly, salivary glands of *dDP* mutant flies that do not have functional dE2F1 and dE2F2 did not display significant variability in the nuclear size (Fig 3A–3C), suggesting that this phenotype is specific to *de2f1b* and not a general consequence of deregulating E2F function.

**Fig 4:**
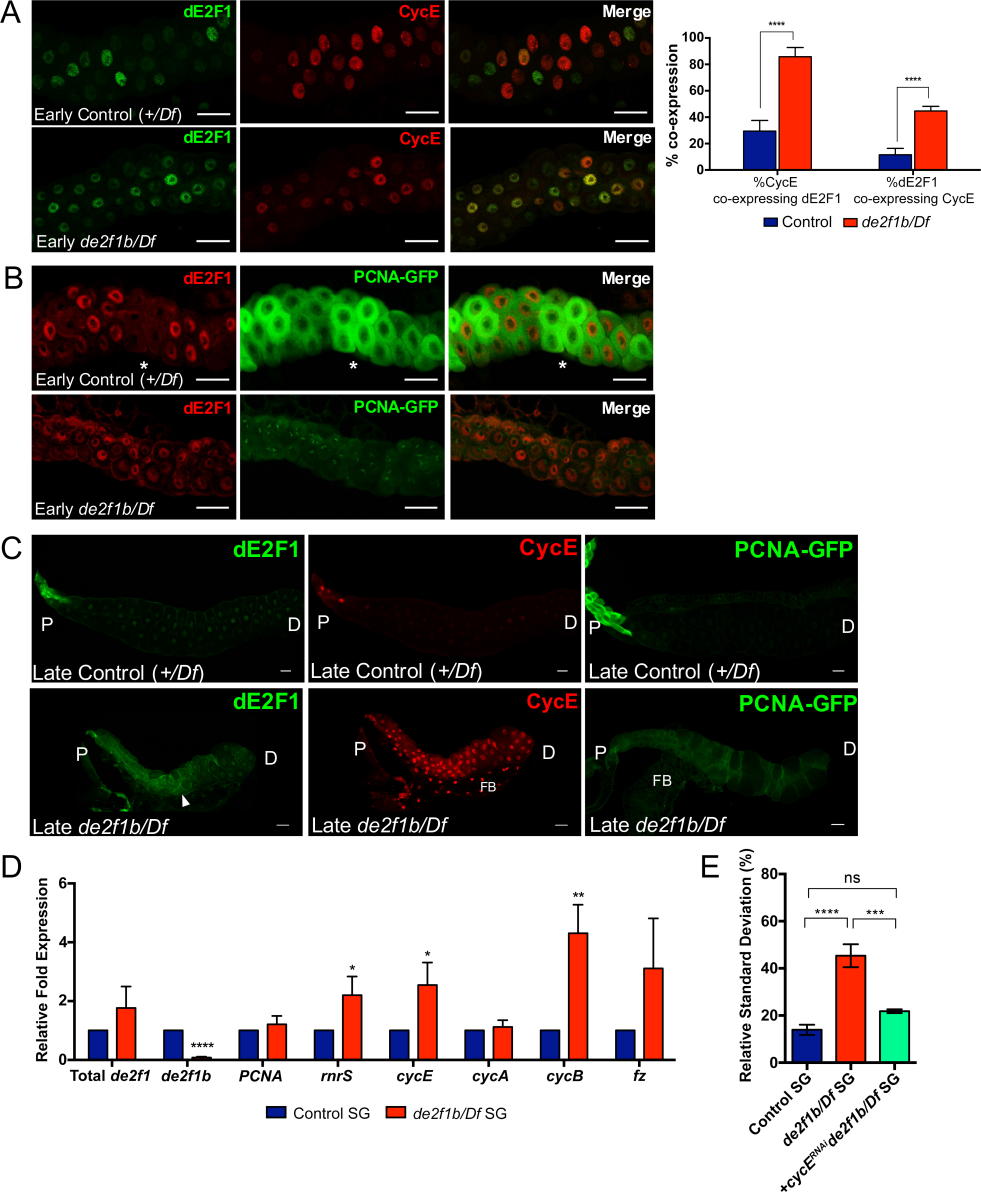
Oscillation of Cyclin E and E2F target gene expression is deregulated in *de2f1b* mutant salivary glands. (A) Salivary glands of control and *de2f1b* mutant early third instar larvae (80–85 hr AEL) are stained with anti-dE2F1 (green) and anti-Cyclin E (CycE, red). Bar graph indicates quantification of the percentages of CycE and dE2F1 expressing cells that co-express dE2F1 and CycE, respectively, in control and *de2f1b* mutant salivary glands. **** = p≤ 0.0001 using two-tailed t-test. (B) Salivary glands of control and *de2f1b* mutant early (80–85 hr AEL) third instar larvae expressing PCNA-GFP (green) are stained with anti-dE2F1 (red). The region where high PCNA-GFP is observed with low dE2F1 is marked by an asterisk. (C) Salivary glands of control and *de2f1b* mutant late third instar larvae (105–110 hr AEL) are stained with anti-Cyclin E (CycE). In addition, dE2F1 activity is monitored using PCNA-GFP, which is a GFP reporter of a well-known E2F target gene, PCNA. Cytoplasmic accumulation of dE2F1 in *de2f1b* mutants is marked by an arrowhead. (D) Relative expression levels of E2F target genes between control and *de2f1b* mutant late (105–110 hr AEL) third instar larval salivary glands are determined by RT-qPCR. Values represent the mean of triplicated biological replicates and error bars represent s.d. * = p ≤ 0.05; ** = p ≤ 0.01; **** = p≤ 0.0001 using two-tailed t-test. (E) Averages of the relative standard deviation of nuclear area from control, *de2f1b* mutant, and *de2f1b* mutant knocking down *cycE* using *heatshock-Gal4* and *UAS-cycE*^*RNAi*^. Values represent the mean of triplicated biological replicates and error bars represent s.d. P values represent: ns = p>0.05; *** = p≤0.001; **** = p≤0.0001 calculated using one-way ANOVA. Scale bars for all salivary glands represent 50 μm. P: proximal end. D: distal end. FB: Fat body.

Endocycle progression in the salivary gland requires dE2F1-dependent periodic expression of CycE [25]. In G1 phase of the cell cycle, dE2F1 accumulates to promote timely expression of CycE at the G1 to S phase transition. During S phase when CycE level is high, dE2F1 is targeted for ubiquitin-dependent degradation by CRL4^CDT2^ [26]. Consequently, CycE and dE2F1 expression are coupled to the cell cycle and largely show a mutually exclusive pattern of expression in early-stage (80–85 hr AEL) L3 salivary glands, when cells are actively cycling ([26] and Fig 4A upper panel). This stereotypic pattern of CycE and dE2F1 expression is disrupted in early-stage *de2f1b* mutant salivary glands. dE2F1 is more broadly expressed and cells expressing extremely high levels of CycE are mostly absent in *de2f1b* mutant salivary glands (Fig 4A lower panel). In addition, more cells in *de2f1b* mutant salivary glands co-express CycE and dE2F1. While only 30% of CycE and 12% of dE2F1 expressing cells co-express the other protein in control salivary glands, these numbers increase to 85% and 45% respectively in *de2f1b* mutant salivary glands (Fig 4A). We also compared the expression pattern of dE2F1 and a dE2F1 activity reporter, PCNA-GFP (Fig 4B). The PCNA-GFP reporter expresses GFP under the control of a region of the *PCNA* promoter that contains well-characterized E2F-binding sites [27]. Interestingly, PCNA-GFP shows a pattern of expression that is similar to CycE in control salivary glands. PCNA-GFP expression is highest in cells with a low level of dE2F1 and low in cells with a high level of dE2F1 (asterisk in Fig 4B upper panel). Two interesting differences are observed in *de2f1b* salivary glands (Fig 4B lower panel). First, the overall intensity of PCNA-GFP is weaker than control although dE2F1 is expressed at a similar level. Second, contrary to what was observed in control salivary glands, PCNA-GFP expression lacks signs of oscillation, being more evenly expressed throughout the salivary gland. Overall, these results indicate that dE2F1b is required for strong activation and periodic expression of its target genes during early L3 salivary gland development.

We next examined late-stage L3 salivary glands (105–110 hr AEL) to determine the overall consequences of the *de2f1b* mutation. In late-stage control salivary glands, dE2F1 is evenly expressed at a lower level than the early stage salivary but higher at the proximal tip where high CycE expression and strong PCNA-GFP activity are also detected (Fig 4C upper panel). In the middle and at the distal tip of the late-stage salivary gland, CycE expression and PCNA-GFP activity is almost undetectable. This indicates that, by this stage, most cells have completed the endocycle and dE2F1 activity is suppressed and limited to the proximal tip of the salivary gland. In late-stage *de2f1b* salivary glands, we observed what seems to be sustained expression of dE2F1, CycE and PCNA-GFP throughout the tissue (Fig 4C lower panel). Importantly, dE2F1 and CycE expression is not limited to the proximal region and visible in the middle and distal tip of *de2f1b* salivary glands (Fig 4C lower panel). Interestingly, we reproducibly detected cytoplasmic dE2F1 signal in many cells of the *de2f1b* salivary gland (arrowhead in Fig 4C). However, the significance of this observation is currently unclear and further studies will be necessary to elucidate its importance. Nevertheless, this result demonstrates that coordinated downregulation of dE2F1 expression and activity is disrupted in late-stage L3 *de2f1b* salivary glands. To support this observation, the expression levels of other dE2F1 target genes were determined by RT-qPCR (Fig 4D). We observed approximately a two-fold increase in G1/S-phase target expression such as *rnrS* and *CycE*. Notably, the total level of *PCNA* is not increased in this assay. The PCNA-GFP shows strong expression in the proximal region of control salivary glands, which is lost in *de2f1b* mutants. Perhaps, higher *PCNA* expression observed in the middle and distal regions of late-stage *de2f1b* salivary glands is balanced out by the loss of strong *PCNA* expression in the proximal region. We also observed a three- to four-fold increase in a number of E2F-regulated G2/M genes such as *cyclin B* (*CycB*) and *fizzy* (*fz*) in *de2f1b* salivary glands. To determine if the sustained expression of dE2F1 target genes is responsible for the salivary gland defects, we reduced the expression level of a key dE2F1 target gene, *cycE*, via RNAi-mediated knockdown. The RNAi-construct was expressed with the *heatshock*-*Gal4* driver without heat shock, to partially suppresses CycE expression (S3 Fig). Strikingly, the variability of nuclear sizes in *de2f1b* salivary glands shown in Fig 3 is significantly suppressed by *cycE* knockdown, indicating that the failure to properly downregulate *cycE* contributes to this phenotype (Fig 4E). Taken together, our results demonstrate that dE2F1b is an isoform of dE2F1 that is necessary for tight regulation of dE2F1 expression and activity during salivary gland development.

**Fig 5:**
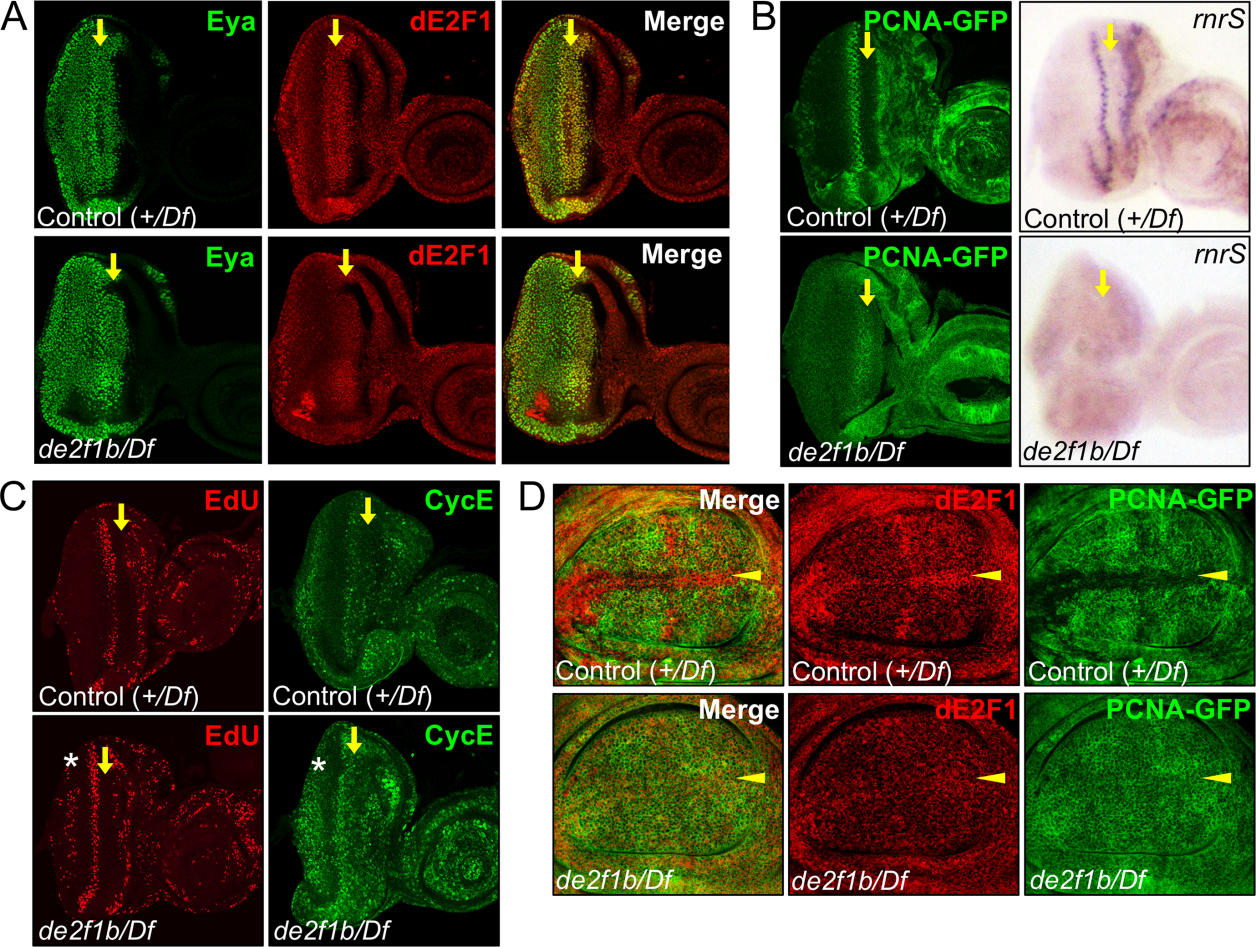
The expression pattern of dE2F1 and its target gene expression is altered in mitotic tissues of *de2f1b* mutants. (A) The expression patterns of dE2F1 (red) in third instar eye imaginal discs of control and *de2f1b* mutant larvae are shown. Eye absent (Eya, green), a nuclear protein, is also visualized in the same eye discs to control for the focal planes of the images. (B) PCNA-GFP activity and the expression pattern of an E2F target gene in third instar eye imaginal discs of the indicated genotypes are determined. For the E2F target gene, a *rnrS* antisense probe is used. (C) Eye imaginal discs of the indicated genotypes are labeled with EdU to visualize S-phase cells (EdU). CycE expression pattern was also determined by anti-CycE antibody. Yellow arrows indicate the position of the morphogenetic furrow. The asterisks indicate the location where ectopic S-phase cells and CycE expression were observed. (D) The expression patterns of dE2F1 and PCNA-GFP in third instar wing imaginal discs of the indicated genotypes are determined. Yellow arrowheads indicate the position of the zone of non-proliferating cell (ZNC) region.

Fig 4C and 4D raise the possibility that dE2F1b plays a repressive function at the late-stage of salivary gland development, either by limiting total level of dE2F1 or by directly repressing target gene expression. However, we cannot exclude the possibility that they are an indirect consequence of incomplete endocycle, which is supported by the DAPI quantification (Fig 3D). To gain better insights into dE2F1b’s function, we analysed *de2f1b* mutant mitotic tissues such eye and wing imaginal discs, which do not have visible developmental defects. Curiously in eye imaginal discs, a number of studies have reported that the dE2F1 protein, despite being an activator, is expressed highest in the morphogenetic furrow where cells are arresting in G1 (Fig 5A upper panel) [28]. Strikingly, in *de2f1b* mutant eye discs, dE2F1 expression in the morphogenetic furrow is greatly reduced (Fig 5A lower panel). In addition, PCNA-GFP expression is ectopically detected in the morphogenetic furrow and the posterior regions of the *de2f1b* eye disc where it is normally repressed (Fig 5B left panel). A similar change in the expression pattern of a dE2F1 target gene, *rnrS*, is also observed (Fig 5B right panel). Interestingly, changes in E2F target gene expression do not greatly alter the pattern of S-phase cells in *de2f1b* eye discs (Fig 5C). However, a reproducible presence of ectopic S-phase cells and CycE expression at the posterior region of *de2f1b* eye discs are observed (Fig 5C asterisks). Notably, we did not observe any ectopic cell death at the same region, suggesting that ectopic S-phase cells observed in *de2f1b* mutant eye discs is not a consequence of simply increasing overall dE2F1 activity (S4 Fig) [28]. In wing imaginal discs, PCNA-GFP expression is normally repressed in the zone of non-proliferating cells (ZNC, Fig 5D arrow heads), where cells are arrested in either G1 or G2 [29]. Similar to the morphogenetic furrow, dE2F1 expression is highest at the ZNC. Strikingly, dE2F1 expression is also greatly reduced and PCNA-GFP is ectopically expressed at the ZNC in *de2f1b* wing discs (Fig 5D). Although direct detection of dE2F1b is required as conclusive evidence, our data suggest that dE2F1b is expressed at developmental stages when cells undergo cell cycle arrest, and that it provides a repressive function on target gene expression in this context.

**Fig 6:**
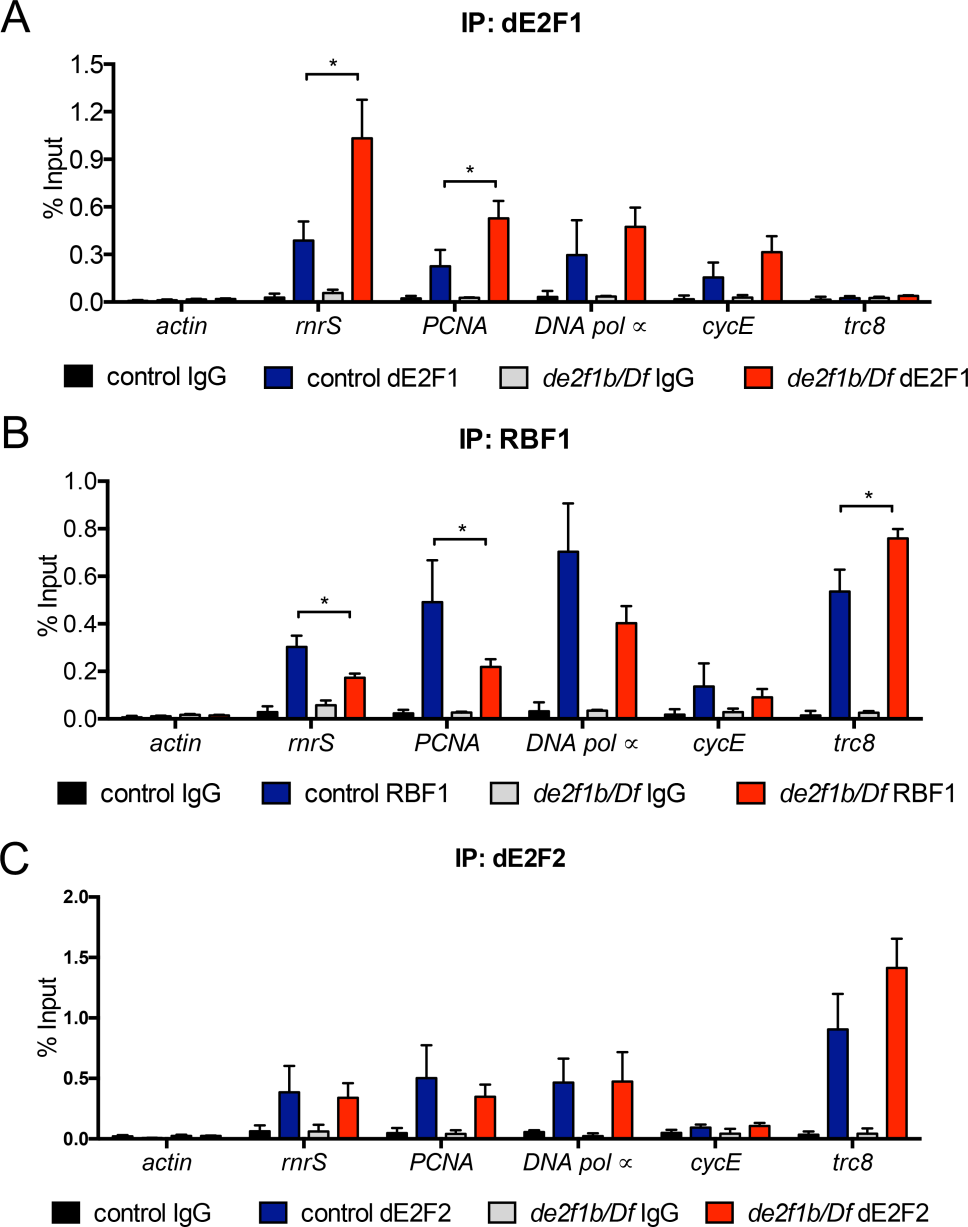
The recruitment of RBF1/dE2F1 to E2F-target genes is altered in *de2f1b* mutants. Chromatin Immunoprecipitation (ChIP) assays were performed to determine the recruitment of dE2F1 (A), RBF1 (B) and dE2F2 (C) to well characterized target genes. Chromatin is isolated from third instar larvae of control and *de2f1b* mutants. Normal rabbit and mouse IgG are used as control where normal rabbit IgG was used for anti-dE2F1 and anti-dE2F2, and normal mouse IgG was used for anti-RBF1. Percent enrichment over the input is calculated from three independent ChIP experiments. *Actin* is used as a negative control of both dE2F1 and RBF1 ChIP. *Trc8* is a dE2F2-specific target gene and a positive control for RBF1 ChIP. Values represent the mean of triplicated biological replicates and error bars represent s.d. P values comparing % input enrichment of control versus *de2f1b* mutant ChIP experiments were calculated using two-tailed t-tests where * = p≤0.05.

To gain molecular insights into how dE2F1b mediates its repressive function, we determined the promoter occupancy of dE2F1 and RBF1, a negative regulator of dE2F1. Recruitment of total dE2F1 and RBF1 to known target genes was quantified between control and *de2f1b* mutant larvae using chromatin immunoprecipitation (ChIP) coupled with qPCR. Anti-dE2F1 ChIP revealed increased recruitment of dE2F1 to S-phase genes such as *rnrS*, and *PCNA* promoters in *de2f1b* mutant compared to control larvae (Fig 6A). Contrary to dE2F1, anti-RBF1 ChIP using the same chromatin extracts showed an overall decrease in the recruitment of RBF1 to the same set of target genes (Fig 6B). Importantly, RBF1 recruitment to a previously identified dE2F2-specific gene, *trc8*, is largely unchanged if not increased in *de2f1b* mutants, indicating that the decrease in RBF1 recruitment is specific to dE2F1 target genes (Fig 6B) [30]. A potential explanation is that dE2F1, whose expression is increased in *de2f1b* mutants (Fig 2E), competes away dE2F2 that normally forms a stable complex with RBF1. However, dE2F2 ChIP shows that dE2F2 recruitment is unaltered in *de2f1b* mutant larvae, indicating that the changes in dE2F1 and RBF1 recruitment are dE2F2-independent (Fig 6C). These observations demonstrate that the lack of dE2F1b promotes dE2F1 recruitment, presumably dE2F1a, and decreases RBF1 recruitment to target promoters, providing a possible molecular explanation of how dE2F1b negatively regulates gene expression.

**Fig 7:**
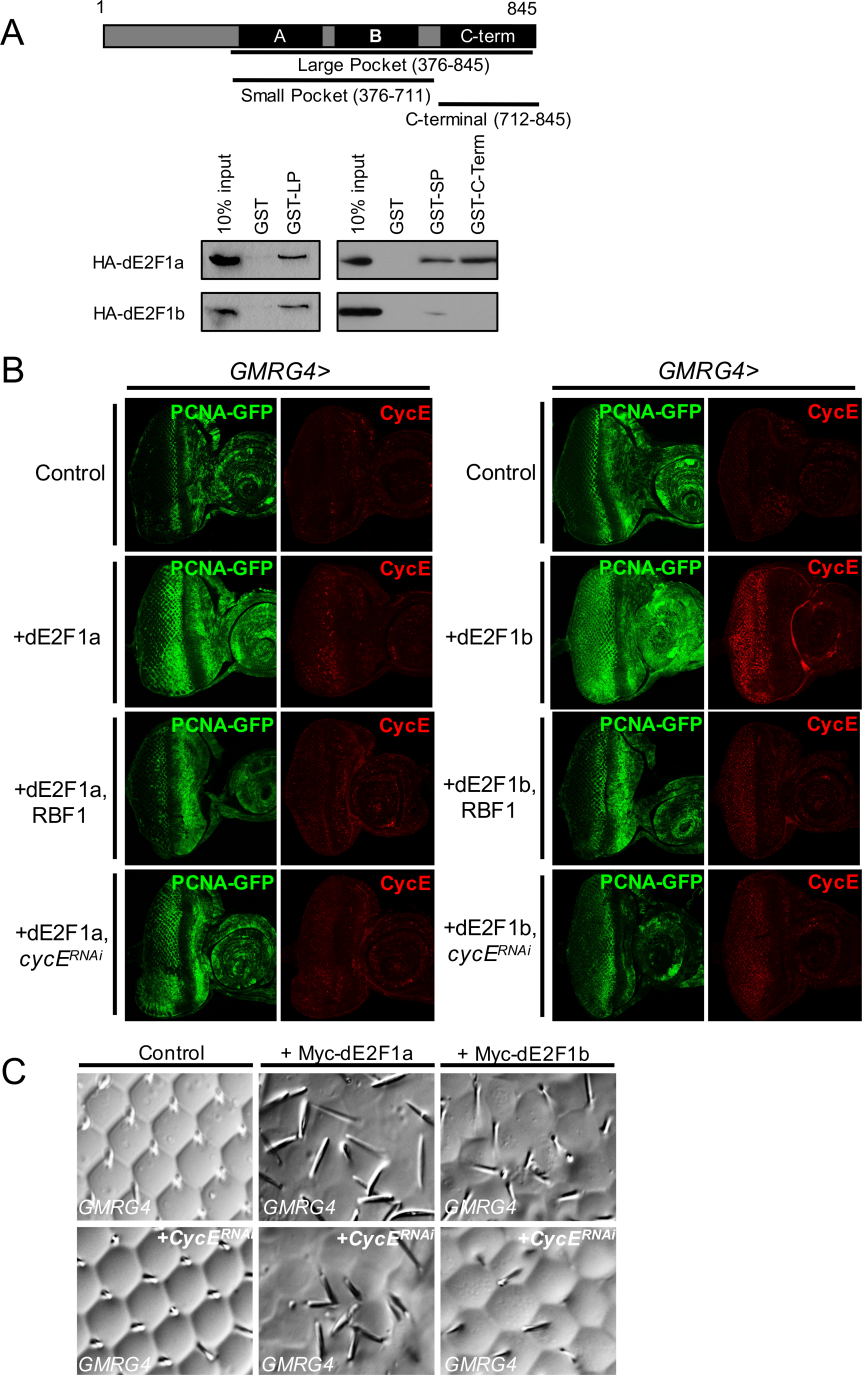
The effect of dE2F1b deregulation is mediated by cyclin E. (A) A schematic of different domains of RBF1 protein and the GST-fusion constructs used in the GST pull-down assay, “Large Pocket” (LP), “Small Pocket” (SP) and C-term are shown. These GST-fusion proteins are used to pull down HA-tagged dE2F1a or dE2F1b that are transiently expressed in S2 tissue culture cells. Anti-HA is used to visualize the transfected protein. A GST alone construct is used as a negative control and 10% of the input is used a loading control. (B) Myc-tagged dE2F1a or dE2F1b is expressed the Drosophila eye using an eye-specific GMR-Gal4 driver (GMRG4). Control eye discs are shown in upper panel. The effects of dE2F1a or dE2F1b overexpression alone (second panel), together with RBF1 (third panel), and together with cycERNAi (bottom panel) on the PCNA-GFP reporter activity and CycE expression are shown. (C) The adult eye morphology is visualized by the nail polish imprinting technique (see Materials and Methods). dE2F1a or dE2F1b is either expressed alone (upper panel) or together with an RNAi construct targeting cycE (lower panel).

Two functional domains of activator E2Fs physically interact with RB family proteins [16–18]. The transactivation (TA) domain interacts with the “pocket” domain of RB, which consists of the A and B domains of RB family proteins, and the MB domain interacts with the C-terminal domain of RB family proteins (Fig 7A). Because the *de2f1b*-specific exon alters the amino acid sequence of the MB domain, we tested if dE2F1a and dE2F1b differentially interact with RBF1 through GST pull-down assays. We made three GST-fusion constructs (Fig 7A), Large Pocket (LP), which includes A, B and C-terminal domains, Small Pocket (SP), which include A and B domains without the C-terminal domain, and C-terminal domain alone (C-term). Not surprisingly, LP pulls down both dE2F1a and dE2F1b expressed in S2 cells since both isoforms contain the same TA domain (Fig 7A). However, C-term only pulls down dE2F1a and not dE2F1b, indicating that the 16-amino acid insertion interferes with this interaction (Fig 7A). Moreover, the SP reproducibly fails to pull down dE2F1b as efficiently as dE2F1a. These results suggest that the 16-amino acid insertion alters the way that dE2F1 interacts with RBF1.

Because dE2F1a and dE2F1b differentially interact with RBF1, we examined if RBF1 can equally affect dE2F1a- and dE2F1b-dependent transcription. For this task, dE2F1a or dE2F1b was overexpressed using an eye-specific GAL4 driver, *GMR-Gal4* (*GMRG4*), and PCNA-GFP was used to monitor their activities. Interestingly, while overexpression of Myc-tagged dE2F1a or dE2F1b in the eye using the *GMRG4* driver results in similar levels of expression (S5 Fig), dE2F1b is more efficient at activating PCNA-GFP than dE2F1a (Fig 7B, second panel). dE2F1b can ectopically activate PCNA-GFP in the entire posterior region of the eye disc while the effect of dE2F1a overexpression is limited to several ommatidial rows posterior to the morphogenetic furrow. We also observed a similar effect on CycE expression, showing a stronger induction by dE2F1b than by dE2F1a. Importantly, the fact that dE2F1b is a better activator of transcription than dE2F1a likely explains why PCNA-GFP expression is weaker in early L3 *de2f1b* salivary glands than the control although the overall dE2F1 expression is relatively unchanged (Fig 4B). To determine the effect of RBF1 on dE2F1a- and dE2F1b-dependent transcription, we co-expressed RBF1. RBF1 expression strongly suppresses the ectopic PCNA-GFP and CycE expression induced by dE2F1a as well as dE2F1b (Fig 7B, third panel). These results demonstrate that although dE2F1a and dE2F1b differentially interact with RBF1, their activities can be negatively regulated by RBF1. We next asked if the ability of dE2F1a and dE2F1b to promote transcription is equally affected by CycE. The MB domain-mediated interaction between different E2F and RB family proteins was recently demonstrated to affect how phosphorylation by cyclin dependent kinases (CDKs) disrupt the RB-E2F complex [19]. In addition, we wanted to determine if the increased CycE expression by dE2F1b contributes to its ability in efficiently activating PCNA-GFP. Strikingly, *cycE* knockdown resulted in different consequences on dE2F1b- and dE2F1a-induced PCNA-GFP expression (Fig 7B bottom panel). While the co-expression of *cycE RNAi* has little to no effect on the dE2F1a’s ability to ectopically induce PCNA-GFP expression, it strongly suppresses the dE2F1b-induced expression of PCNA-GFP. Moreover, the dE2F1b-induced adult eye phenotype, but not the dE2F1a-induced eye phenotype, is suppressed by *cycE* knockdown (Fig 7C). Taken together, our results suggest that while both dE2F1a and dE2F1b can activate transcription, dE2F1b’s ability promote transcription is largely dependent on CycE.

## Discussion

Having only one “activator” E2F and one “repressor” E2F, the *Drosophila* RB/E2F network is widely accepted as a streamlined version of the mammalian RB/E2F network. Here, we present an additional level of complexity to the *Drosophila* RB/E2F network. Alternative splicing generates two isoforms of *de2f1*, one of which was previously uncharacterized. Through molecular complementation tests and generating an isoform-specific mutant, we demonstrated that dE2F1b is an important regulator of the cell cycle during development, particularly in the salivary gland. Curiously, we also found evidence to suggest that dE2F1b may have a repressive function on target gene expression in a context-specific manner.

The widely-used *de2f1a* cDNA was isolated more than two decades ago from an eye imaginal disc cDNA library [31]. Given that the *de2f1b* contributes to only about 10% to the total *de2f1* transcript in the eye disc (Fig 1C), it is not surprising that it is *de2f1a* and not *de2f1b* that was originally cloned. Our molecular complementation test (Fig 1D and 1E) indicates that dE2F1a and dE2F1b may have distinct roles during development. It will be interesting to revisit some previous experiments where the existence of dE2F1b was not considered. For example, a dominant modifier screen on an dE2F1b-induced eye phenotype may identify a different set of genes from those identified with dE2F1a [32]. Our data also suggest that RNA splicing is an important step of regulating dE2F1 function. Interestingly, splicing factors have been identified as regulators of dE2F2 [33]. Identification of factors involved in the alternative splicing of *de2f1* will likely reveal previously unappreciated regulators of the cell cycle. Notably, the difference between the two *de2f1* isoforms lies in the MB domain, which is associated with member-specific functions in mammals [14]. Perhaps, *Drosophila* evolved to express E2Fs with distinct functions through alternative splicing rather than having multiple E2F genes.

The *de2f1b*-specific mutant described in this study has an increased level of *de2f1* transcript (Fig 2E). One possible explanation for this observation is that dE2F1b normally limits overall *de2f1* expression. At this point, it is difficult to test this hypothesis since a dE2F1b specific antibody does not exist. An alternative explanation is that exon 3b, which was deleted in our study, contains important regulatory sequences that control *de2f1* expression at the level of transcription or RNA processing. When we designed the *de2f1b*-specific mutant, we decided to remove the entire exon 3b sequence instead of mutating splicing donor and acceptor sites. We reasoned that this approach would cleanly remove the *de2f1b* isoform without causing aberrant alternative splicing. However, if indeed exon 3 contains regulatory sequences, this approach may have resulted in an unintended effect on *de2f1* expression. While we did monitor dE2F1 protein expression throughout our study, it will be important to engineer a new set of *de2f1b* alleles by specifically targeting splicing donor and acceptor sites. These alleles can help determine if the *de2f1b* mutant defects described in this study are indeed specifically caused by the lack of the *de2f1b* isoform.

Perhaps it is not surprising that *de2f1b* mutants have defects in salivary glands. Several studies have previously demonstrated that deregulated dE2F1 activity results in endocycle defects. *de2f1*^*su89*^ mutants, which have a point mutation that weakens the interaction with RBF1, have defects in endocycling tissues in a *de2f2* mutant background [34]. Furthermore, *de2f1*^*i2*^ mutants, which have a truncation in the C-terminal transactivation domain, are female sterile due to defects in ovarian follicle and nurse cells, which are also endocycling cells [35, 36]. It is worth noting that *de2f1*^*su89*^ and *de2f1*^*i2*^ are hypermorphic and hypomorphic mutants of *de2f1* respectively, and they both have endocycle defects. Clearly, dE2F1 activity has to be tightly controlled in these tissues. We found evidence to show that dE2F1b is not only a potent activator of transcription (Fig 7B), but also capable of carrying out a repressive function (Fig 5). In addition, we also showed that dE2F1b can efficiently induce CycE expression (Fig 7B, second panel). We speculate that the dual function of dE2F1b on gene expression and its ability to strongly induce CycE makes dE2F1b an ideal dE2F1 isoform that governs salivary gland development.

An unanticipated finding from this study is that while dE2F1b is a potent activator of transcription, it can provide a repressive function in a context-specific manner. The *de2f1b*-specific exon alters amino acid sequences in the MB domain that make direct contact with the C-term of RB family proteins (S6 Fig). Our GST pull-down experiments showed that while both dE2F1a and dE2F1b are capable of binding to LP of RBF1, dE2F1b fails to interact with C-term of RBF1 (Fig 7A). This result suggests that dE2F1a and dE2F1b differentially interact with RBF1 and may form different protein complexes. It is plausible that the MB domain of dE2F1b specifically interacts with proteins that can provide a repressive function. Indeed, the MB domain of activator E2Fs is shown to mediate protein-protein interactions in a member-specific manner [15]. Another interesting aspect of the MB domain-mediated interaction is that it influences the way that CDKs affect the stability of the RB-E2F complex [19]. Therefore, it is possible that while both dE2F1a and dE2F1b can interact with RBF1, dE2F1b is able to form a stable repressive complex with RBF1 when the CDK activity is low. Indeed, the repressive function of dE2F1b is observed in the morphogenetic furrow of the eye disc and in ZNC of the wing discs where cells are arrested in G1 or G2 (Fig 5). Moreover, dE2F1b-dependent transcription can be efficiently suppressed by *cycE* depletion (Fig 7B).

One of the clear differences between the two dE2F1 isoforms is their relationship with CycE. dE2F1b more strongly induces CycE expression than dE2F1a and is more sensitive to *cycE* knockdown (Fig 7B and 7C). These results suggest that dE2F1b function is more tightly linked to the cell cycle than dE2F1a. If this is true, what then is the role of dE2F1a during development? It is conceivable that dE2F1a carries out cell cycle-independent functions associated with activator E2Fs. For example, mammalian E2F1 is shown to specifically regulate cell death genes and its ability to silence repetitive sequences is clearly cell cycle-independent [20]. Interestingly, the MB domain of E2F1 interacts with the C-term of RB in the GST pull-down assay while the MB domains of E2F2 and E2F3 do not [19]. Similarly, we observed that the MB domain of dE2F1a interacts with C-term of RBF1 while the MB domain of dE2F1b does not (Fig 7A). It is possible that dE2F1a has a cell cycle-independent function similar to those associated with E2F1 in mammals. It will be interesting to generate a *de2f1a* specific mutant to precisely determine its function during development.

While dE2F1b is a canonical E2F by structure, its repressive function may be analogous to the function of atypical E2Fs in mammals, E2F7 and E2F8. First, both dE2F1b and E2F7/8 negatively regulate E2F target genes (Fig 2E and Fig 4D and [37–40]). Second, one of the key genes regulated by dE2F1b and E2F7/8 is an activator E2F (Fig 2E and [41, 42]). Third, the tissues that are primarily affected by the inactivation of dE2F1b and E2F7/8 are endoreplicating tissues [43, 44]. E2F7/8 knockout mice have ploidy defects in trophoblasts and hepatocytes. The functional similarity between dE2F1b and E2F7/8 suggests that although the exact mechanism may be different, the interplay between members of E2F proteins may be evolutionarily conserved.

## Materials and methods

### Fly strains

All fly strains and crosses were maintained at 25°C with standard cornmeal medium. *w*^*1118*^ and *yw* flies were used as controls. The following alleles were used: For *de2f1* mutants, *de2f1*^*rm729*^ [22] alleles were crossed to the *Df(3R)Exel6186* deficiency allele which lacks the entire *de2f1* gene locus (Exelis collection at the Harvard Medical School). *dDP*^*a3a1*^ and *dDP*^*a4a3*^ alleles [7] were crossed together to generate dDP mutants. *PiggyBac* transposase stock (#8285) was obtained from the Bloomington Stock Center for removal of the ScarlessDsRed cassette for *de2f1b* mutant generation. For overexpression and rescue experiments, the following GAL4 lines were obtained from the Bloomington Drosophila Stock Center: *Ubi-Gal4*, *GMR-Gal4*, and *hs-Gal4* (Bloomington, IN, USA). For knock-down of CycE, *UAS-CycE-RNAi* was obtained from the Vienna Drosophila Resource Center (Vienna, Australia). *PCNA-GFP* was obtained from Dr. Duronio [27]. *UAS-FM-dE2F1a* and *UAS-FM-dE2F1b* overexpression constructs were generated by using the *Drosophila* Gateway collection (Drosophila Genomic Resource Center). The entry clones, pENTR-dE2F1 and pENTR-dE2F1b, were generated then recombined into the pTFM destination vector to be randomly integrated into the *Drosophila* genome. Minimum of 10 independent transgene lines were screened to identify lines with similar levels of expression.

### Generation of *de2f1b* mutant allele

The *de2f1b* mutant allele was generated in two steps. First, *de2f1b*-specific exon was replaced by ScarlessDsRed cassette by Wellgenetics, Taiwan. In short, two independent guide RNAs (gRNAs), CRISPR Target Site 1[PAM]: CTCTTTTGCTGCCGAGCGGT[CGG] and CRISPR Target Site 2[PAM]: ACGTTCAAATTGAAGGGGAG[CGG], were used to target regions flanking the *de2f1b*-specific exon, were cloned into the pDCC6 vector [45]. For homology-directed repair (HDR), a pUC57-Kan donor plasmid was used containing the upstream and downstream homology arms of *de2f1* and a ScarlessDsRed cassette flanked by *PiggyBac* transposon ends to facilitate screening [45]. The gRNA plasmids and donor plasmid were co-injected into isogenized *w*^*1118*^ embryos. Hatched G0 flies were crossed to balancer stocks. Balanced stocks were screened using a fluorescent microscope and PCR to confirm the insertion of the ScarlessDsRed cassette into the *de2f1* gene locus in the right orientation. We received the selected *de2f1b-DsRed* mutant lines and used *PiggyBac* transposition to excise the cassette. The *de2f1b* specific mutation was validated by sequencing of genomic DNA and cDNA isolated from the mutant flies.

### Rescue experiment

All molecular complementation crosses were conducted in the *de2f1*^*729*^/*Df(3R)Exel6186* mutant background and UAS-FM-dE2F1a, UAS-FM-dE2F1b, and UAS-FM-dE2F1a + UAS-FM-dE2F1b (recombined) transgenes were driven by *Ubiquitin-Gal4*. To determine rescue efficiency, viability was assessed by counting the total number of L3 larvae, pupae, pharate adults, and adult flies that eclosed. For each rescue cross, minimum of 30 virgin females were use. The rescue was confirmed using RT-PCR specific to endogenous transcript by targeting 5’ UTR (S1B Fig). For every cross, the predicted Mendelian frequency of the rescue genotype is 1 in 9. This was obtained by first determining the expected frequency of the rescued genotype based on the Mendelian ratio, which is 1 in 16. When taking into account the homozygous Balancers that die at the embryonic stage, the predicted Mendelian survival frequency becomes 1 in 9. For each cross, the number of progeny counted is as follows: control n = 710, dE2F1a rescue n = 625, dE2F1b rescue n = 545, dE2F1a+dE2F1b rescue n = 515, *de2f1* mutant n = 572, and 2XdE2F1a rescue n = 469. Percentage survival at L3, for each genotype is the following: control = 100%, dE2F1a rescue = 14.4%, dE2F1b rescue = 19.8%, dE2F1a+dE2F1b rescue = 100%, *de2f1* mutant = 0%, and 2XdE2F1a rescue = 23.1%. Percentage survival at pupal stage for each genotype is the following: control = 100%, dE2F1a rescue = 8.6%, dE2F1b rescue = 13.2%, dE2F1a+dE2F1b rescue = 100%, *de2f1* mutant = 0%, and 2xdE2F1a rescue = 23.1%. Lastly, percentage survival at pharate stage (adult for control) is the following: control = 99%, dE2F1a rescue = 0%, dE2F1b rescue = 0%, dE2F1a+dE2F1b rescue = 83.89%, *de2f1* mutant = 0%, and 2xdE2F1a rescue = 0%. These values are used to plot a Kaplan-Meier curve to visualize percentage survival (Fig 1D).

### Immunostaining and EdU labeling

The following antibodies were used: rabbit anti-dE2F1 (1/100, generous gift from N. Dyson, Massachusetts General Hospital), mouse anti-Myc (1/200, Developmental Studies Hybridoma Bank (DSHB), mouse anti-Eya (1/200, DSHB), rat anti-ELAV (1/200, DSHB), rabbit anti-cleaved Dcp-1 (1/100, Cell Signaling), goat anti-CycE (1/200, Santa Cruz sc15903), goat anti-GFP conjugated to FITC (1/100, Abcam ab6662), and secondary antibodies coupled to fluorescent dyes (1:500, Jackson Immunoresearch). For Immunostaining, third instar imaginal discs and salivary glands were dissected in PBS and immediately fixed in 4% formaldehyde in PBS for 20 minutes at room temperature with the exception of tissues subjected to anti-dE2F1 staining that were fixed for 30 minutes on ice. Fixed tissues were then washed with 0.3% PBST (0.3% TritonX-100 in 1XPBS) and 0.1% PBST (0.1% TritonX-100 in 1XPBS). Samples were incubated with appropriate amount of primary antibody in 0.1% PBST and 1%BSA overnight. Samples were then washed with 0.1% PBST, incubated in secondary antibody in 0.1% PBST and 1% BSA for 2 hours, followed by several washes in 0.1% PBST prior to mounting. To visualize S-phase cells, Ethynyl-2’-Deoxyuridine (EdU) cell proliferation assay (Invitrogen C10339) was used according to the manufacturer’s specifications in third instar eye imaginal discs and salivary glands. DNA was visualized with 0.1 μg/mL DAPI. All salivary glands were appropriately staged as either early (80–85 hours after egg laying, AEL) or late (105–110 hours AEL) L3 salivary glands for immunostainings. Representative images were selected from a minimum of 10 independent tissues that were appropriately labelled.

### Nail polish imprinting

Adult ommatidial structure was examined by using the nail polish imprinting technique as previously described [46]. In brief, decapitated adult heads were placed in a clear nail polish then quickly removed with the eye side up to dry at room temperature for 1 hour. The nail polish imprint was then carefully peeled off using tungsten needles and mounted for imaging. Minimum of 5 adult heads were analyzed. Image presented depicts the most representative image.

### *In situ* hybridization

Third instar eye imaginal discs were prepared for in situ hybridization as described previously [47]. In brief, the *de2f1* and *rnrS* RNA probes were prepared and anti-DIG antibody conjugated with alkaline phosphatase was used to visualize RNA probes. At least 20 discs were examined and representative images were selected.

### Microscopy

All fluorescently labelled tissues were mounted using a glycerol-based anti-fade mounting medium containing 5% N-propyl gallate and 90% glycerol in 1XPBS. Images were acquired using a laser-scanning Leica SP8 confocal microscope at the Cell Imaging and Analysis Network, McGill University. L3 salivary glands and L3 wing discs were imaged using the 20x/0.7 dry objective. L3 eye discs were imaged using the 40x/1.3 oil immersion objective. Representative images are individual slices from z-stacks. Nail polish imprint of the adult eyes were mounted in 100% glycerol and imaged using the DIC channel of the Zeiss AxioImager Z2. Bright field images of whole larvae, well-fed 5 day old ovaries, salivary glands, and discs subjected to *in situ* hybridization were imaged using the Canon Powershot G10 and Zeiss SteREO Discovery V8 modular stereo microscope with a conversion lens adaptor. All images were processed using Fiji (http://fiji.sc/Fiji).

### Quantification of salivary gland nuclear size

Nuclear area of DAPI-stained nuclei in the distal region of third instar larval salivary glands was measured using the particle analysis tool in Fiji. A minimum of 35 nuclei that were in the correct focal plane were required for the size distribution analysis. For each genotype, three salivary glands were used for quantification. Each salivary gland nuclear size distribution was plotted on a box and whiskers plot according to its genotype. Average standard deviation was calculated for each salivary gland then averaged to represent a mean value.

### Quantification of DAPI intensity in salivary glands

For DAPI quantification, DAPI-stained salivary glands were imaged using Leica SP8 confocal microscope where each salivary gland was scanned through using 1 μm Z-stack steps. To quantify DNA content, Fiji was utilized where for every Z-stack image, maximum intensity projection was created then mean fluorescence intensity was used as DNA content values. Three late staged (105–110 hr AEL) salivary glands were examined for analysis.

### Quantification of dE2F1 and CycE co-expression

Co-expression was quantified using the colocalization analysis tool using Imaris software (Bitplane). 3D colocalization analysis was performed for three independent salivary glands for wildtype and *de2f1b* mutant early stage (80–85 hr AEL) salivary glands that were scanned through using 1 μm Z-stack steps. For each channel representing either dE2F1 or CycE expression, consistent threshold values were utilized. Values presented represent percentage of dE2F1 expressing cells above threshold that have co-localized with CycE or percentage of CycE expressing cells above threshold that co-localized with dE2F1.

### RNA extraction and cDNA synthesis

For RT-qPCR, RNA was extracted from different developmental stages including 0-6hr Embryos, L3 whole larvae, 48 After Puparium Formation (APF) pupae, and adults (5 days old; 3 females and 2 males). RNA was also collected from L3 eye and wing imaginal discs, and L3 salivary glands of the appropriate genotype using the miRNAeasy Mini Kit (Qiagen). To eliminate genomic contamination, the RNA was treated with RNAse-free DNase I. 500ng of RNA was used to synthesize cDNA using the DyNAmo cDNA Synthesis Kit (Finnzymes) with random hexamer primers.

### Reverse transcriptase quantitative PCR (RT-qPCR)

Gene expression was measured using the DyNAmo Flash SYBR Green qPCR Kit (ThermoScientific) with the Bio-Rad CFX 96 Real-Time System and C1000 Thermal Cycler. RT-qPCR was performed as described by [21]. For all RT-qPCR experiments, the data was normalized using two housekeeping genes, *rp49* and *β-tubulin* and to the control (wildtype whole larvae or tissue). Each experiment consisted of experimental triplicates and overall, three biological replicates were averaged for the representative figure in this paper. All primers were designed using Primer3 (Whitehead Institute for Biomedical Research, http://Frodo.wi.mit.edu/primer3/). All primers were tested and run on an 8% acrylamide gel to ensure that there is no genomic contamination in our results. The following primers were used in this paper:

*rp49* forward: TACAGGCCCAAGATCGTGAAG
*rp49* reverse: GACGCACTCTGTTGTCGATACC
*β-tubulin* forward: ACATCCCGCCCCGTGGTC
*β-tubulin* reverse: AGAAAGCCTTGCGCCTGAACATAG
Total *de2f1(de2f1*_*2-3*_*)* forward: CAGCACCACCACCAAAATC
Total *de2f1(de2f1*_*2-3*_*)* reverse: ACTGCTAGCCGTATGCTTCTG
*de2f1b(de2f1*_*3b-4*_*)* forward: AACCGCTCCCCTTCAATTTG
*de2f1b(de2f1*_*3b-4*_*)* reverse: GTTGGTCAACGGATGCAGTC
*rnrS* forward: AATGGCGTCCAAGGAAAAC
*rnrS* reverse: ACATCTTGCGAACGTTGTTG
*PCNA* forward: AAGCCACCATCCTGAAGAAG
*PCNA* reverse: CGACACATGGGAGTTGTCC
*cycE* forward: GTTTGTGCAAACCTCACAGC
*cycE* reverse: AACAGCGTAAAGCCATCTCC
*cycB* forward: GCTGCCGATTCACTTCCTTC
*cycB* reverse: CAGCTGCAATCTCCGATGG
*fz* forward: TCCGTCTCGTACAACACCAG
*fz* reverse: CTGACGGGTGACAACGAGTA

### Absolute quantification of gene expression

Absolute quantification of gene expression for total *de2f1* and *de2f1b* transcripts was performed with RNA isolated from different developmental stages and L3 tissues (Fig 1B). Standard curves for total *de2f1* and *de2f1b* specific primer pairs were generated using specific amounts of plasmid DNA containing *de2f1b* sequences. Log_10_ of plasmid copy numbers of a serial dilution were plotted against the experimentally determined qPCR quantification cycle (Cq) values of each dilution. The linear regression from each plot was then used to derive an equation to calculate the copy number / unit of cDNA. For each sample, an average Cq value of three independent experiments consisting of biological triplicates was used to determine the copy number / unit of cDNA (representing 25ng of RNA). Error bars indicate standard error of the mean (s.e.m.).

### Chromatin immunoprecipitation (ChIP)—Quantitative PCR (qPCR)

Chromatin was collected from 80 third instar larvae from appropriate genotypes. Control or *def1b* mutant larvae were homogenized in Buffer A1 (50mM KCl, 15mM Nacl, 4mM MgCl_2_, 15mM HEPES pH7.6, 0.5% TritonX-100, 0.5mM DTT, protease inhibitor cocktail, Roche), cross-linked in 1.8% Formaldehyde followed by addition of 225mM Glycine, followed by three washes in Buffer A1 and one wash with lysis buffer without SDS (140mM NaCl, 15mM HEPES pH7.6, 1mM EDTA, 0.5mM EGTA, 1% TritonX-100, 0.5mM DTT, 0.1% DOC, protease inhibitor cocktail). DNA was sheared to 500-100bp fragments by sonicating for 3X30 second intervals with two-minute breaks in Elution buffer 1 (140mM NaCl, 15mM HEPES pH7.6, 1mM EDTA, 0.5mM EGTA, 1% TritonX-100, 0.5mM DTT, 0.1% DOC, 0.1% SDS, 0.5% N-Lauroylsarcosine, protease inhibitor cocktail) for the collection of chromatin. Chromatin was immunoprecipitated using rabbit anti-dE2F1 or, mouse anti-RBF1 [8]. Rabbit and mouse IgG were used as non-specific antibodies. Chromatin-antibody complexes were pulled down with Protein A/G-Sepharose Beads and washed four times in lysis buffer (140mM NaCl, 15mM HEPES pH7.6, 1mM EDTA, 0.5mM EGTA, 1% TritonX-100, 0.5mM DTT, 0.1% DOC, 0.05% SDS, protease inhibitor cocktail), once in LiCl buffer (10mM Tris pH8, 250mM LiCl, 1mM EDTA, 0.5% DOC, 0.5%NP-40) and once in TE pH8 buffer. DNA was eluted from antibody-bound A/G beads using Elution 2 buffer (1%SDS, 100mM NaHCO_3_) then treated with RNase A and Proteinase K. DNA was precipitated and cleaned using two rounds of phenol-chloroform extraction followed by a chloroform extraction then resuspended in ddH_2_O. Presented data are averages of triplicated ChIP experiments which consisted of experimental duplicates followed by quantitative real time PCR reactions. Presented data for target loci enrichment is represented by percentage of input chromatin not subjected to immunoprecipitation. All primers were designed by Primer3 and primers used for ChIP-qPCR analysis are the following:

*act88F* forward: CCAACTCAAATCGCTTCGAG
*act88F* reverse: CGCACTCACACACCTTTTAG
*PCNA* forward: TTTCACATCCCTATCCCGCT
*PCNA* reverse: ACTGATGACGGGCTTAAGAAT
*cycE* forward: ATCTCCGTCCCTCCCCTAG
*cycE* reverse: GTGATCGCTTTGGAACCGAA
*DNA polymerase α* forward: CAGGTCGGATTTCCCGCCAAAATA
*DNA polymerase α* reverse: GTGACCAGGGATGGAGGATGATCA
*rnrS* forward: TGACAAGCTGGGAAGCTAAA
*rnrS* reverse: TGACAAGCTGGGAAGCTAAA
*trc8* forward: GGCTGTGACTTTGGGATGAA
*trc8* reverse: ATATCGCCCGTGGCTTTT

### Statistical analysis

Statistical test performed using two-tailed unpaired t-test assuming equal variance unless otherwise stated. For Figs 3C and 4E, statistical testing was performed using one-way ANOVA with Sidak’s multiple comparisons test, comparing mean of each column to every other column. For all statistical analyses, p ≤ 0.05 was considered statistically significant.

### S2 cell transfection and protein extraction

*Drosophila* S2 cell culturing was carried out in Schneider’s medium (Sigma) supplemented with 2mM L-Glut, 50U Penicillin-Streptomycin, 10% heat-inactivated FBS. Cells were passaged 1:5 three days prior to transfection. A transient transfection was performed using the Effectene Transfection reagent kit (QIAGEN) to co-transfect cells with 0.8 μg GFP, and 2.4 μg of either pAFHW-E2F1a or pAFHW-E2F1b. GFP was co-transfected to visualize transfection efficiency. 48 hours post-transfection, cells were washed in 1X PBS, and re-suspended in 250 μL of protein extraction buffer (PEB: 20mM Tris-HCl pH8.0, 137mM NaCl, 10% glycerol, 1% TritonX-100, 2mM EDTA, protease inhibitor cocktail). Cells were lysed by vortexing 7 times (15s/vortex with 5min breaks) and crude protein lysate was obtained by centrifuging at 12000 rpm for 20min at 4°C.

### Plasmid generation and transformation into BL21 cells

GST-RBF1-large pocket (LP), GST-RBF1-small pocket (SP), and GST-RBF1-C-terminus (C-term) constructs were generated using the pGEX-5x-1 vector (GE Healthcare Life Sciences) backbone digested with BamH1/Xho1 (NEB). LP, SP, and C-term segments of RBF1 was generated by using cDNA of full length RBF1. BamH1 and Xho1 sites were added to primer ends for successful ligation into vector. Primer sequences used for cloning is the following:

BamH1-RBF1 LP/SP forward–TTTCTGGATCCCCGTCCGCAACGC
Xho1-RBF1-SP reverse–TTCCCCTCGAGGTAGTCGATGACGAA
BamH1-RBF1 C-term forward–CTTCGGGATCCTAAACGTAACTCCGGACGTGA
Xho1-RBF1 LP/C-term reverse–CCCCTCGAGCTATGTCTCGTG

Successfully ligated vectors with appropriate inserts were then transformed into BL21 cells (NEB). Empty pGEX-5x-1 vector was used as a GST-alone control which was transformed into DH5-alpha cells (Thermo Fisher).

### Protein purification and GST-pulldown

10mL culture of BL21 cells expressing GST-RBF1-LP, GST-RBF1-SP, GST-RBF1-C-term (Fig 7A) and GST alone were grown overnight in a 37°C shaker at 2500 rpm. The overnight culture was diluted and grown in the 37°C shaker until an OD_600_ of 0.7. Next, 1 μL of 100mM IPTG was added per mL culture. Induction for GST-RBF1 C-term was performed by incubating in the 37°C shaker for 3 hrs. For GST-RBF1-SP and GST-RBF1-LP, which have higher molecular weights, induction was performed overnight at room temperature (22°C). Cells were pelleted, re-suspended in 3mL of PEB, and lysed by sonication. Lysate was centrifuged and supernatant was transferred to fresh tubes where 120 μL of glutathione agarose beads (50% slurry, Thermo Fisher) were added. The tubes were then incubated for 2hrs at 4°C with agitation. Beads were washed 3X in PEB and re-suspended with PEB to make a 50% slurry. For each pull-down assay, 150 μg of S2 cell extracts transfected with dE2F1a or dE2F1b and sepharose beads bound with approximately 2 μg GST-tagged RBF1-LP/SP/C-term or GST alone were used. Beads were incubated overnight at 4°C with agitation, and washed 3X with PEB. Samples were then loaded on an 8% polyacrylamide gel for western blot analysis.

### Western blot

All blots were blocked with 10% skim milk powder in 0.1% PBS-Tween20. For the GST-pulldown, 1/1000 dilution of anti-HA rat antibody (abcam) was used followed by 1:2000 dilution of anti-rat HRP (GE Healthcare). To measure dE2F1a and dE2F1b transgene overexpression efficiencies, 10 L3 eye discs were dissected from control, GMRG4>FM-dE2F1a, and GMRG4>FM-dE2F1b overexpression groups. Anti-Myc (1/1000 dilution, DSHB) and Anti- β-tubulin (1/1000 dilution, DSHB) were used as primary antibodies followed by anti-mouse HRP (1/2000, GE Healthcare).

## Supporting information

S1 Fig Confirmation of the *de2f1* mutant rescue by RT-PCR specific to endogenous *de2f1* transcripts. (A) A representative rescued *de2f1* mutant pharate adult is shown. (B) To confirm the rescue by the transgenic constructs, the absence of endogenous *de2f1* transcripts in the rescues flies is determined by RT-PCR targeting the 5’ UTR region. (C) Table indicating average days after egg laying (AEL) when 3^rd^ instar larvae (L3) from indicated rescue crosses were observed. (D) A survival curve showing *de2f1*^*-/-*^ rescue using 2 copies of the dE2F1a transgene.
(TIF)

S2 Fig Genomic sequences of *de2f1b* mutants covering the deleted exon. (A) Genomic DNA from control (*yw*) and *de2f1b* mutant (*de2f1b/Df*) flies are sequenced and compared to the annotated sequences covering the exon unique to *de2f1b*. Capital letters indicate the 3b exon. Note that the *de2f1b* mutants precisely lack the exon and flanking splicing acceptor and donor sites. *ttaa* in *de2f1b* mutants is the footprint produced by PiggyBac Transposition (see Materials and Methods). (B) Bright field images of ovaries from well-fed five days old control (*yw*) and *de2f1b* (*de2f1b/Df*) adult females are shown. Scale bar represents 0.5 mm. (C) Full length coding sequence and translation product of *de2f1* obtained from *de2f1b* mutants.
(TIF)

S3 Fig Reducing the level of CycE using an RNAi construct. Salivary glands from control, *de2f1b* mutants, and *de2f1b* mutants expressing dsRNAi targeting *cycE* are shown. The *cycE*^*RNAi*^ construct is expressed by a *heatshock-Gal4* driver. An anti-CycE antibody was used to determine the effect on CycE levels. Even in the absence of heat shock, the overall level and the number of cells with an intense CycE staining are reduced in the presence of heat shock Gal4 driver due to the leakiness of the driver. Scale bar represents 100 μm.
(TIF)

S4 Fig Eye discs of *de2f1b* mutants do not have ectopic cell death. (A) Control eye discs and eye discs overexpressing dE2F1a (GMRG4>dE2F1a) and dE2F1b (GMRG4>dE2F1b) are shown. Apoptotic cells and S-phase cells are visualized by a cell death marker, Cleaved *Drosophila* Caspase-1 (Dcp-1, green) and EdU (red) respectively. The asterisks show apoptotic cells and arrow head show S-phase cells that are induced by overexpression of dE2F1a or dE2F1b. (B) Eye discs of control and *de2f1b* mutants are immunostained for a neuronal marker (ELAV, blue) and a cell death marker, Cleaved Drosophila Caspase-1 (Dcp-1, green).
(TIF)

S5 Fig Overexpression of dE2F1a and dE2F1b in the *Drosophila* eye. (A) Immunoblot (upper panel) and immunostaining (lower panel) with anti-Myc compare the expression levels of dE2F1a and dE2F1b. 20 pairs of the discs for each genotype were used for the Immunoblot and β-tubulin is used as a loading control. The GMR-Gal4 driver is used to express Myc-tagged dE2F1a or dE2F1b in the *Drosophila* eye.
(TIF)

S6 Fig Multiple sequence alignment of the Marked Box (MB) domain of fruit flies and human. The MB domain sequences from the two *Drosophila* dE2F1 isoforms, dE2F1a and dE2F1b, and all six canonical E2Fs from human, E2F1 to 6, are aligned. Highlighted in green are four amino acids that are identified to be important for specific interactions between E2F and RB family proteins (18). Red letters indicate the amino acid sequences coded by the *de2f1b*-specific exon. Asterisks indicate the amino acids in the E2F5 MB domain that makes contact with the C-terminal domain of p107 (18). Arrowheads indicate the amino acids in the E2F1 MB domain that makes contact with the C-terminal domain of pRB (17).
(TIF)
