# Supplementary figures and images for: An alternatively spliced form affecting the Marked Box domain of Drosophila E2F1 is required for proper cell cycle regulation
Source: PLoS Genet. 2018 Feb 8;14(2):e1007204. doi: 10.1371/journal.pgen.1007204 (PMC5821395; doi:10.1371/journal.pgen.1007204)

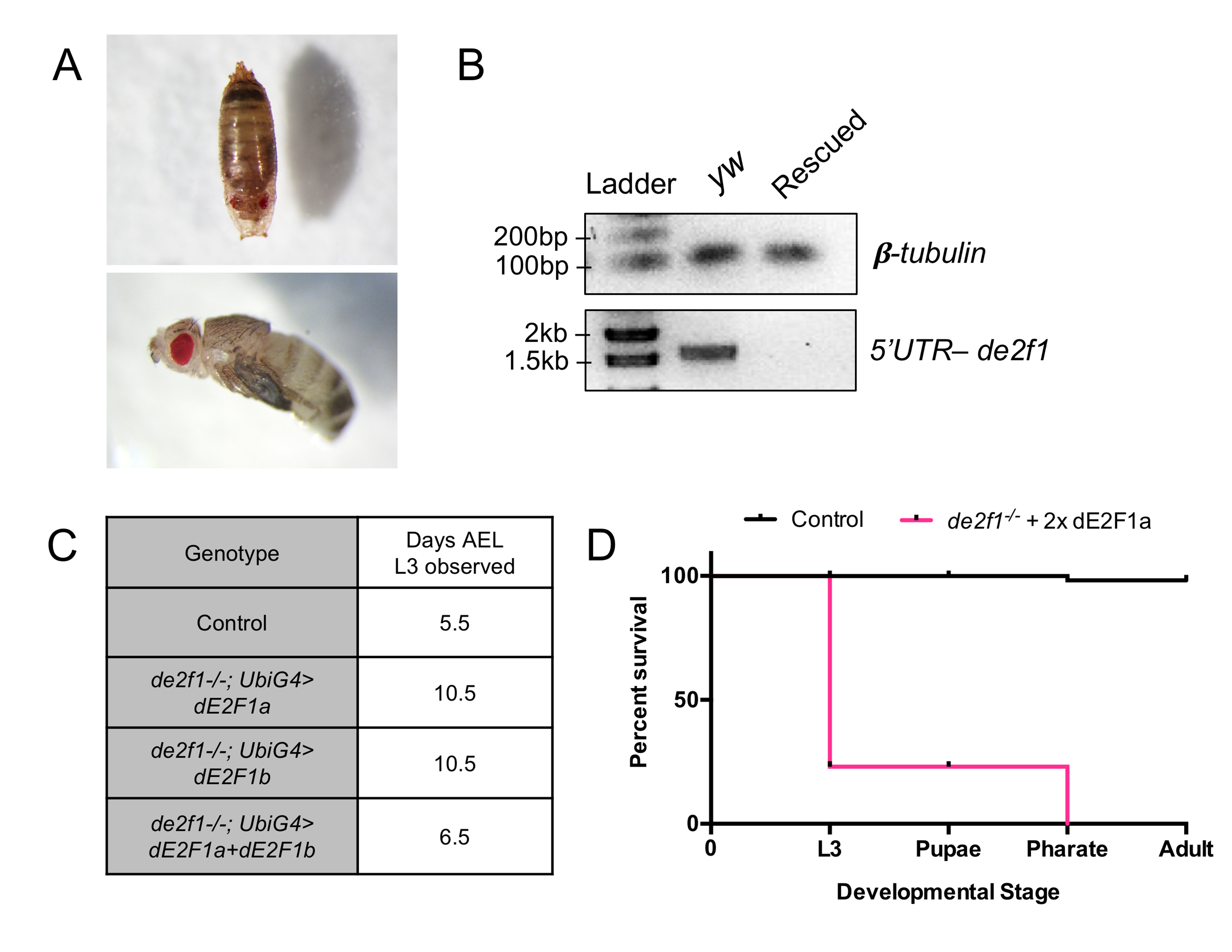

Supplement: S1 Fig — (A) A representative rescued de2f1 mutant pharate adult is shown. (B) To confirm the rescue by the transgenic constructs, the absence of endogenous de2f1 transcripts in the rescues flies is determined by RT-PCR targeting the 5’ UTR region. (C) Table indicating average days after egg laying (AEL) when 3rd instar larvae (L3) from indicated rescue crosses were observed. (D) A survival curve showing de2f1-/- rescue using 2 copies of the dE2F1a transgene. (TIF) [file pgen.1007204.s001.tif]

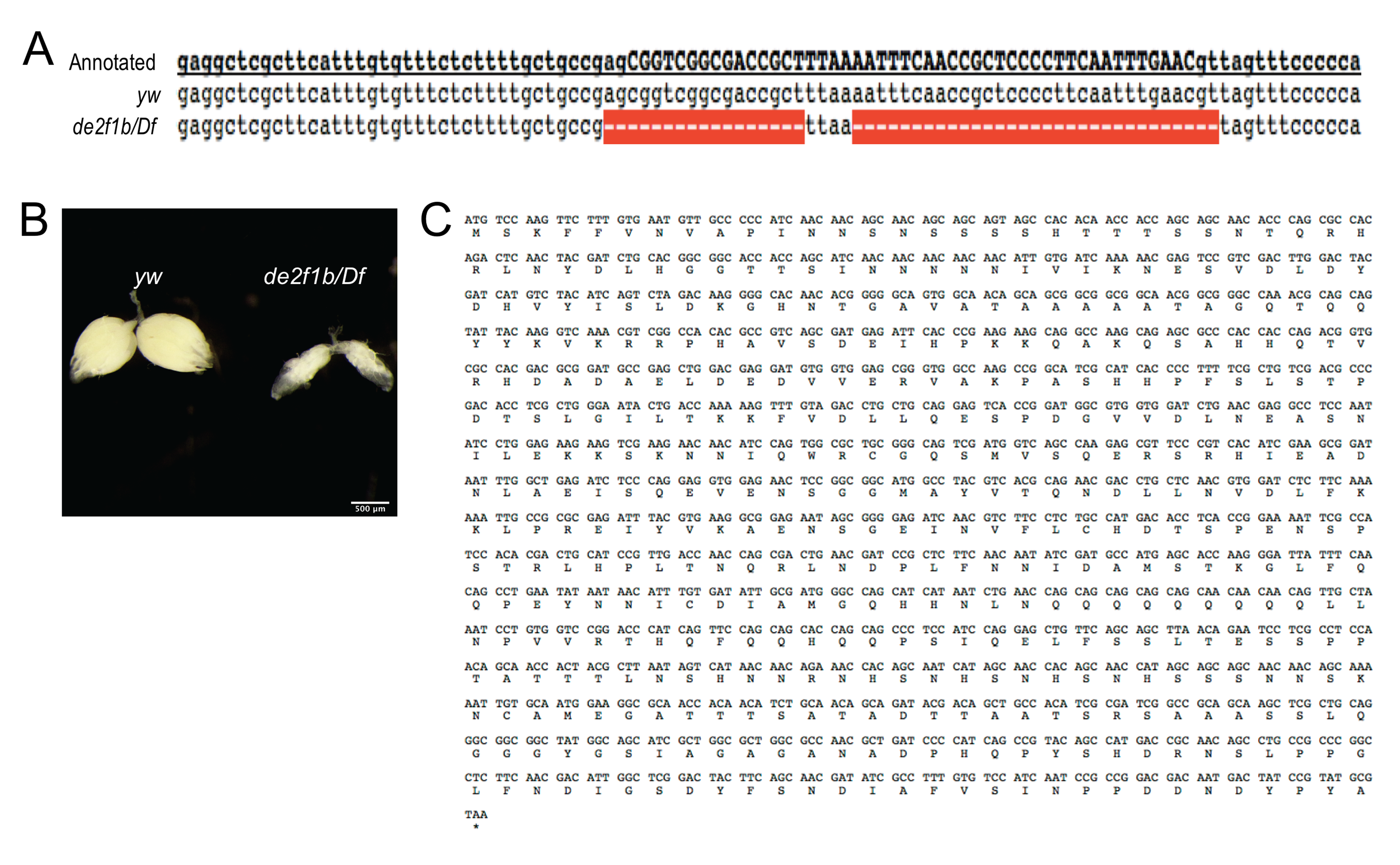

Supplement: S2 Fig — (A) Genomic DNA from control (yw) and de2f1b mutant (de2f1b/Df) flies are sequenced and compared to the annotated sequences covering the exon unique to de2f1b. Capital letters indicate the 3b exon. Note that the de2f1b mutants precisely lack the exon and flanking splicing acceptor and donor sites. ttaa in de2f1b mutants is the footprint produced by PiggyBac Transposition (see Materials and Methods). (B) Bright field images of ovaries from well-fed five days old control (yw) and de2f1b (de2f1b/Df) adult females are shown. Scale bar represents 0.5 mm. (C) Full length coding sequence and translation product of de2f1 obtained from de2f1b mutants. (TIF) [file pgen.1007204.s002.tif]

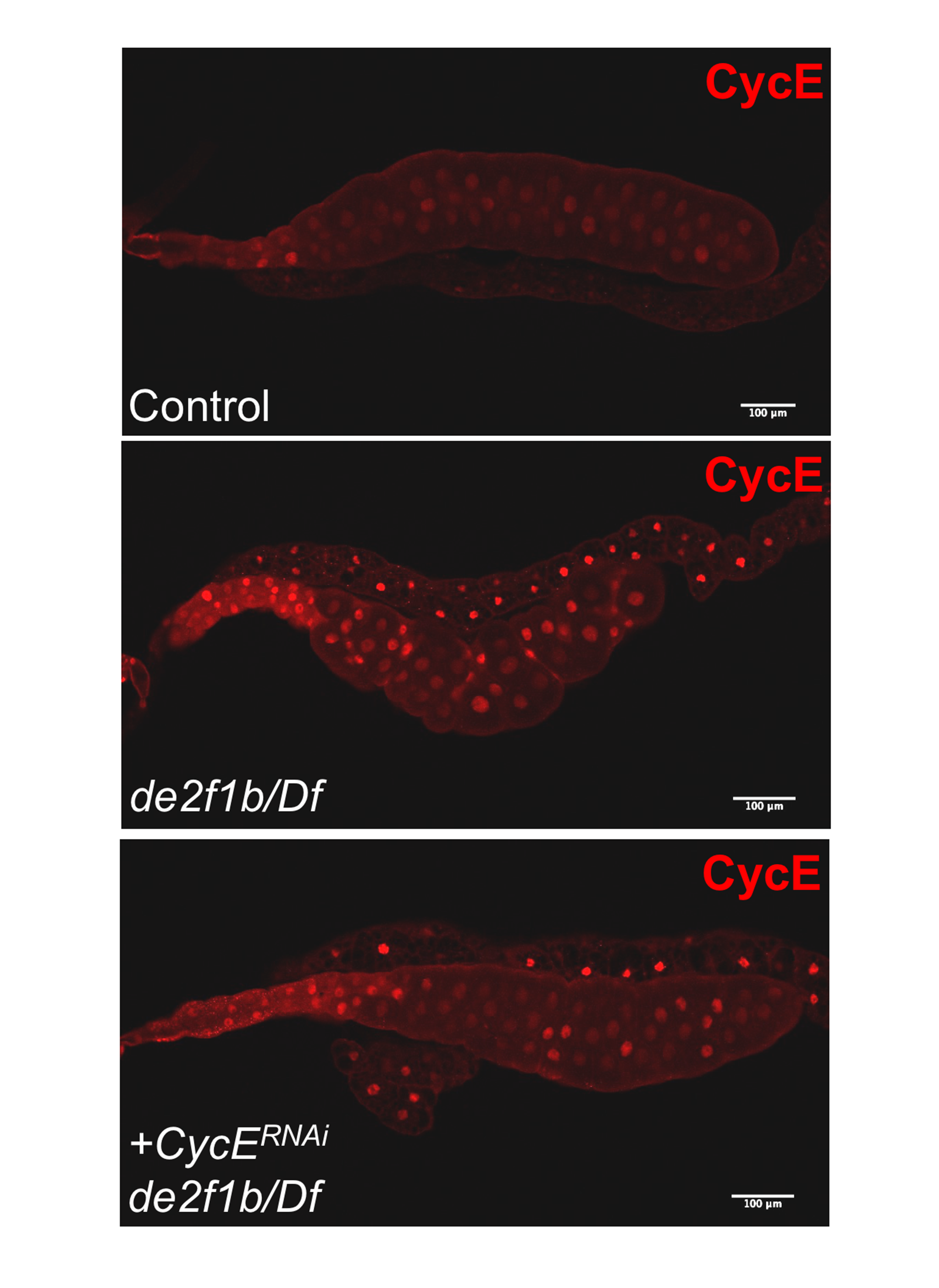

Supplement: S3 Fig — Salivary glands from control, de2f1b mutants, and de2f1b mutants expressing dsRNAi targeting cycE are shown. The cycERNAi construct is expressed by a heatshock-Gal4 driver. An anti-CycE antibody was used to determine the effect on CycE levels. Even in the absence of heat shock, the overall level and the number of cells with an intense CycE staining are reduced in the presence of heat shock Gal4 driver due to the leakiness of the driver. Scale bar represents 100 μm. (TIF) [file pgen.1007204.s003.tif]

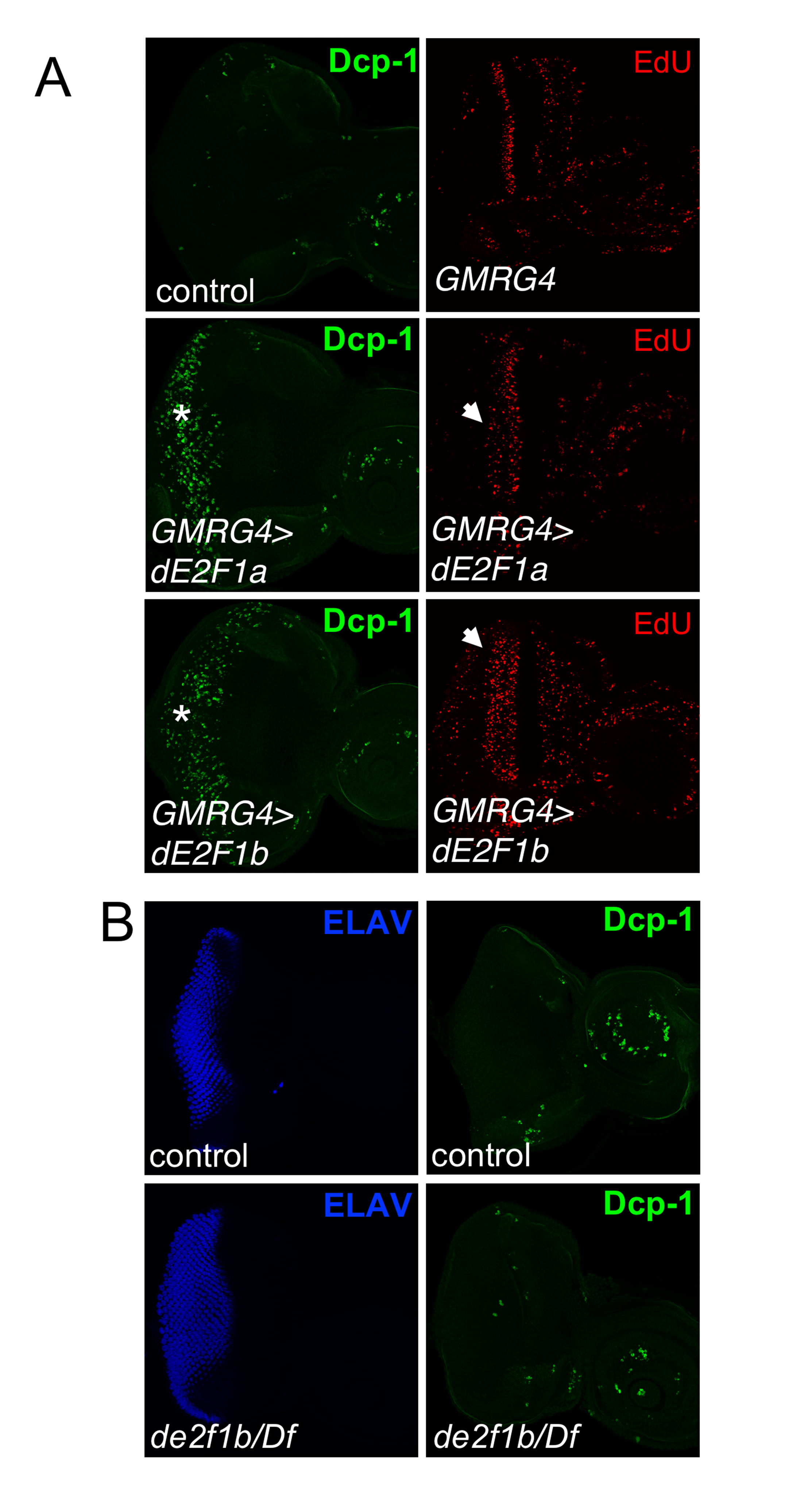

Supplement: S4 Fig — (A) Control eye discs and eye discs overexpressing dE2F1a (GMRG4>dE2F1a) and dE2F1b (GMRG4>dE2F1b) are shown. Apoptotic cells and S-phase cells are visualized by a cell death marker, Cleaved Drosophila Caspase-1 (Dcp-1, green) and EdU (red) respectively. The asterisks show apoptotic cells and arrow head show S-phase cells that are induced by overexpression of dE2F1a or dE2F1b. (B) Eye discs of control and de2f1b mutants are immunostained for a neuronal marker (ELAV, blue) and a cell death marker, Cleaved Drosophila Caspase-1 (Dcp-1, green). (TIF) [file pgen.1007204.s004.tif]

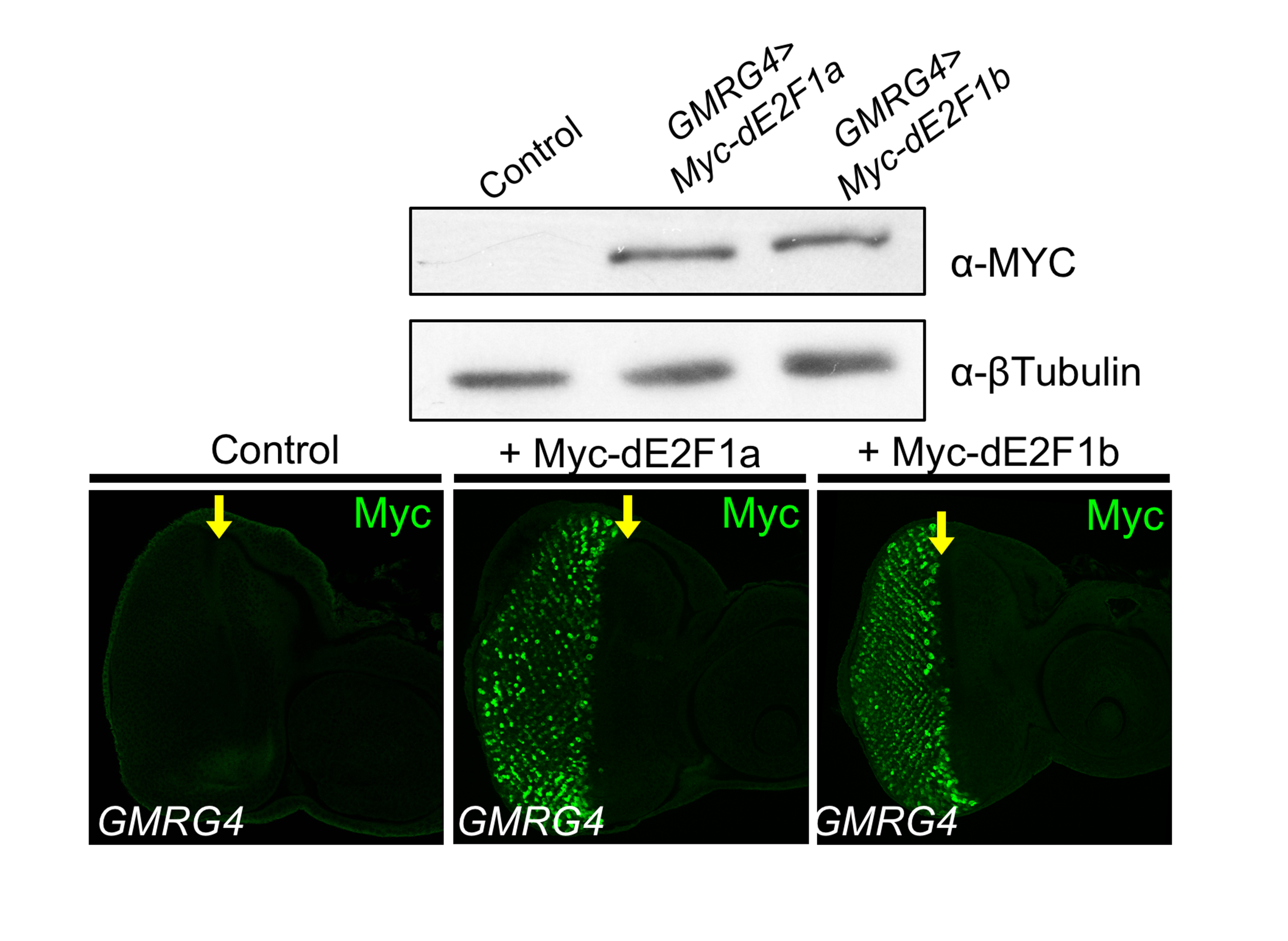

Supplement: S5 Fig — (A) Immunoblot (upper panel) and immunostaining (lower panel) with anti-Myc compare the expression levels of dE2F1a and dE2F1b. 20 pairs of the discs for each genotype were used for the Immunoblot and β-tubulin is used as a loading control. The GMR-Gal4 driver is used to express Myc-tagged dE2F1a or dE2F1b in the Drosophila eye. (TIF) [file pgen.1007204.s005.tif]

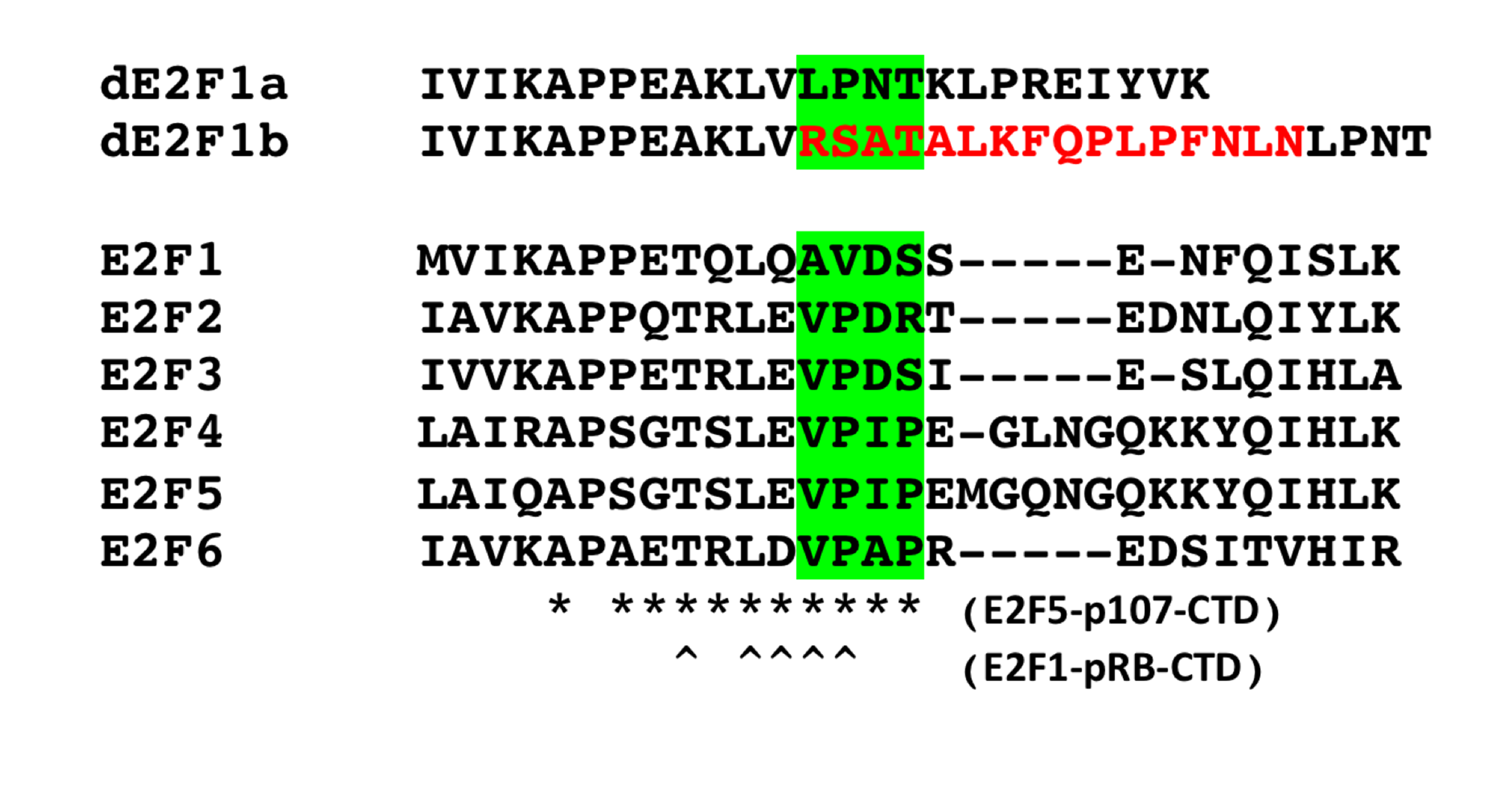

Supplement: S6 Fig — The MB domain sequences from the two Drosophila dE2F1 isoforms, dE2F1a and dE2F1b, and all six canonical E2Fs from human, E2F1 to 6, are aligned. Highlighted in green are four amino acids that are identified to be important for specific interactions between E2F and RB family proteins (18). Red letters indicate the amino acid sequences coded by the de2f1b-specific exon. Asterisks indicate the amino acids in the E2F5 MB domain that makes contact with the C-terminal domain of p107 (18). Arrowheads indicate the amino acids in the E2F1 MB domain that makes contact with the C-terminal domain of pRB (17). (TIF) [file pgen.1007204.s006.tif]
